# Neurocognitive Outcomes at Age 2 Years After Neonatal Hypoglycemia in a Cohort of Participants From the hPOD Randomized Trial

**DOI:** 10.1001/jamanetworkopen.2022.35989

**Published:** 2022-10-11

**Authors:** Taygen Edwards, Jane M. Alsweiler, Greg D. Gamble, Rebecca Griffith, Luling Lin, Christopher J. D. McKinlay, Jenny A. Rogers, Benjamin Thompson, Trecia A. Wouldes, Jane E. Harding

**Affiliations:** 1 Liggins Institute, The University of Auckland, Auckland, New Zealand; 2 Department of Paediatrics: Child and Youth Health, The University of Auckland, Auckland, New Zealand; 3 Newborn Services, Auckland City Hospital, Auckland, New Zealand; 4 Kidz First Neonatal Care, Counties Manukau Health, Auckland, New Zealand; 5 School of Optometry and Vision Science, Waterloo, Canada; 6 Center for Eye and Vision Research, Hong Kong; 7 Department of Psychological Medicine, The University of Auckland, Auckland, New Zealand

## Abstract

**Importance:**

Neonatal hypoglycemia is common, but its association with later neurodevelopment is uncertain.

**Objective:**

To examine associations between neonatal hypoglycemia and neurocognitive outcomes at corrected age 2 years.

**Design, Setting, and Participants:**

Exploratory cohort analysis of the Hypoglycaemia Prevention With Oral Dextrose (hPOD) randomized clinical trial was conducted. The trial recruited participants from January 9, 2015, to May 5, 2019, with follow-up between January 26, 2017, and July 31, 2021. Infants were recruited from 9 maternity hospitals in New Zealand and assessed at home or in a research clinic. Children born late preterm and at term at risk of neonatal hypoglycemia but without evidence of acute or imminent illness in the first hour after birth were screened and treated to maintain blood glucose concentrations greater than or equal to 47 mg/dL.

**Exposures:**

Hypoglycemia was defined as any blood glucose concentration less than 47 mg/dL, recurrent as 3 or more episodes, and severe as less than 36 mg/dL.

**Main Outcomes and Measures:**

Neurologic examination and tests of development (Bayley III) and executive function. The primary outcome was neurosensory impairment (any of the following: blindness, deafness, cerebral palsy, developmental delay, or executive function total score worse than 1.5 SD below the mean).

**Results:**

A total of 1197 of 1321 (91%) eligible children were assessed at a mean of corrected age 24 months; 616 (52%) were male. Compared with the normoglycemia group, children who experienced hypoglycemia were more likely to have neurosensory impairment (111 [23%] vs 125 [18%]; adjusted risk ratio [aRR], 1.28; 95% CI, 1.01-1.60), particularly if they experienced severe episodes (30 [28%] vs 125 [18%]; aRR, 1.68; 95% CI, 1.20-2.36), but not recurrent episodes (12 [19%] vs 125 [18%]; aRR, 1.06; 95% CI, 0.63-1.80). The risk of cognitive, language, or motor delay was similar between groups, but children who experienced hypoglycemia had lower Bayley-III composite cognitive (adjusted mean difference [aMD], −1.48; 95% CI, −2.79 to −0.18) and motor scores (aMD, −2.05; 95% CI, −3.30 to −0.79).

**Conclusions and Relevance:**

In children born at risk of hypoglycemia but otherwise well, those who experienced neonatal hypoglycemia were more likely to have neurosensory impairment at corrected age 2 years, with higher risks after severe episodes. Further research is required to determine causality.

## Key Points

**Question:**

Is neonatal hypoglycemia associated with neurocognitive outcomes in otherwise well late preterm and full-term term neonates born at risk of hypoglycemia?

**Findings:**

In this cohort study of 1197 participants in a randomized clinical trial, the prevalence of neurosensory impairment at 2 years was significantly higher in those who experienced hypoglycemia than in those who did not (23% vs 18%). The risk was greater in children who experienced severe episodes (28%).

**Meaning:**

The findings of this study suggest that children who experienced neonatal hypoglycemia are at increased risk of neurosensory impairment at corrected age 2 years, even if screened and treated to maintain blood glucose concentrations greater than or equal to 47 mg/dL.

## Teaser

This cohort study examines neurodevelopment in children at corrected age 2 years who experienced neonatal hypoglycemia.

## Introduction

Neonatal hypoglycemia is common.^1,2^ Symptomatic or prolonged hypoglycemia causes brain injury and death,^3,4,5^ but the outcomes associated with brief transitional hypoglycemia are less clear.^5^

Neonatal hypoglycemia is associated with increased risk of preschool executive dysfunction and visual-motor impairment, but neurodevelopmental impairment has only been reported at ages 6 to 11 years,^6^ perhaps because the skills affected by hypoglycemia are best detected in older children.^7^ However, most studies were small or of poor quality and included neonates at risk of neonatal illnesses associated with both hypoglycemia and poorer neurodevelopmental outcomes, making it difficult to distinguish their separate effects.^5,6,8,9^ We examined neonatal hypoglycemia and neurocognitive outcomes at corrected age 2 years in a large cohort of late preterm and full-term neonates born at risk of hypoglycemia but without evidence of acute neonatal illness.

## Methods

### Study Design and Participants

This was an exploratory cohort analysis of the Hypoglycaemia Prevention With Oral Dextrose (hPOD) multicenter, randomized clinical trial and its 2-year follow-up, reported elsewhere.^10,11,12^ Eligible infants were recruited from 9 hospitals in New Zealand between January 9, 2015, and May 5, 2019. The primary outcome of the hPOD trial was admission to the neonatal intensive care unit (NICU) for 4 hours or longer^11^ and, of the follow-up study, was neurosensory impairment at corrected age 2 years.^12^ The hPOD trial registration number was ACTRN12614001263684.

### Inclusion Criteria

Inclusion criteria were as follows: age less than 1 hour after birth, 1 or more risk factors for hypoglycemia (maternal diabetes, small [birthweight <2.5 kg or <10th centile], large [birthweight >4.5 kg or >90th centile], or preterm [35-36 weeks’ gestation]), birthweight greater than or equal to 2.2 kg, gestation greater than or equal to 35 weeks, no apparent indication for NICU admission, and mother intending to breastfeed.

Eligible children of caregivers who provided written consent were assessed at corrected age 2 years between January 26, 2017, and July 31, 2021. Travel costs were reimbursed but no additional financial compensation was provided. The study was approved by the New Zealand Health and Disability Ethics Committee and follows the Strengthening the Reporting of Observational Studies in Epidemiology (STROBE) reporting guideline.

### Procedures

Neonates were randomized to prophylactic buccal dextrose, 40%, or placebo gel (0.5 mL/kg) 1 hour after birth and breastfed.^11^ Blood glucose concentration was measured using a glucose oxidase method 2 hours after birth, and neonates were then screened and treated for hypoglycemia according to hospital practice.^13^

Hypoglycemia was defined as 1 or more episodes of consecutive blood glucose concentrations less than 47 mg/dL (to convert to millimoles per liter, multiply by 0.0555) in the first 48 hours after birth. Severity of hypoglycemia was defined as none (all blood glucose concentrations ≥47 mg/dL), mild (≥1 episode ≥36 mg/dL and <47 mg/dL), and severe (≥1 episode <36 mg/dL). Frequency of hypoglycemia was defined as none, 1 to 2 episodes, and recurrent as 3 or more episodes. Treatment for hypoglycemia usually involved combinations of supplemental feeds, buccal dextrose gel, and intravenous dextrose.

At corrected age 2 years, children underwent neurologic examination and tests of development and executive function administered by trained assessors at a research clinic or at home. Caregivers completed questionnaires about the child’s medical and family history.

Children were assessed using Bayley Scales of Infant and Toddler Development, Third Edition (Bayley-III) cognitive, language, and motor scales (mean [SD], 100 [15]) composite score.^14^ We assigned a score of 49 to children unable to be assessed due to severe delay. The performance-based executive function battery comprised 4 tasks measuring simple inhibition (snack delay task), complex inhibition (fruit Stroop and reverse categorization tasks), and attentional flexibility (multisearch/multilocation task).^15^ The scores of each task (range, 0-6 points; ≤2 indicates low performance) were summed to give an executive function total score (range, 0-24 points). For both assessments, higher scores indicate better performance.

Socioeconomic status was reported using the New Zealand Deprivation Index 2018.^16^ Primary risk factor was prioritized in the following order: maternal diabetes, preterm, small, and large. Maternal ethnicity was self-reported and prioritized according to the New Zealand Standard Classification, as required by our ethics committee.^17^

### Outcomes

The primary outcome was neurosensory impairment, defined as any of the following: blindness (visual acuity <3/60 or >1.3 logMAR), hearing impairment requiring aids, cerebral palsy, developmental delay (Bayley-III cognitive, language, or motor composite score <85), or performance-based executive function total score more than 1.5 SD below the cohort mean.

Secondary outcomes were components of the primary outcome and severity. Moderate or severe neurosensory impairment was defined as any of the following: blindness, hearing impairment requiring aids, moderate or severe cerebral palsy (not walking yet or permanently nonambulant), or moderate or severe developmental delay. Developmental delay was defined as none (Bayley-III cognitive, language, and motor composite score ≥85), mild (Bayley-III cognitive, language, or motor composite score, 70-84), and moderate or severe (Bayley-III cognitive, language, or motor composite score <70).

### Statistical Analysis

Analyses were prespecified, unless otherwise indicated. We imputed scores of children missing 1 or more task scores on the performance-based executive function battery, using the cohort mode to calculate an executive function total score. We did not impute other data because it was assumed missing not at random. We did not adjust for multiplicity because this study was exploratory. We compared categorical data using Fisher exact or χ^2^ tests and continuous data using independent-sample *t* tests or Wilcoxon rank sum tests.

For primary analyses, we compared the risk of the primary and categorical secondary outcomes between children who experienced 1 or more episodes of hypoglycemia (hypoglycemia group) and children with no episodes (normoglycemia group) using log-binomial generalized linear regression adjusted for hospital site, socioeconomic decile,^17^ primary reason for risk of hypoglycemia, and multiple births as fixed effects. We compared secondary continuous outcomes using identity-normal generalized linear regression and ordinal outcomes using generalized cumulative logit regression. We compared none vs mild and none vs severe for severity outcomes using log-binomial generalized linear regression.

For secondary analyses, we compared no episodes of hypoglycemia with mild or severe episodes and 1 or 2 or recurrent episodes, using the same methods as the primary analysis. In a post hoc analysis restricted to children who experienced hypoglycemia, we tested for interactions between frequency of episodes and treatment (buccal dextrose plus feed vs intravenous dextrose) on the risk of neurosensory impairment, moderate, or severe neurosensory impairment, and their components, using adjusted generalized linear modeling.

In subgroup analyses, we compared neurosensory impairment, moderate or severe neurosensory impairment, and their components between children of mothers with diabetes and those with other risk factors for hypoglycemia, and between boys and girls using adjusted generalized linear regression with interaction terms. In sensitivity analyses, we excluded children with postneonatal diagnoses likely to affect the outcome, whose first language was not English, and assessed outside the planned assessment window of corrected ages 23 to 25 months. Because this cohort participated in a randomized trial, we undertook a sensitivity analysis of the primary outcome excluding children randomized to the intervention (prophylactic buccal dextrose gel).

We performed all analyses in SAS, version 9.4 (SAS Institute Inc) using 2-sided tests with 95% CIs and *P* ≤ .05 considered statistically significant. Data are presented as unadjusted risk difference or mean difference (MD), and adjusted risk ratio (aRR) or mean difference (aMD), with 95% CIs.

## Results

There were 1359 children recruited to the hPOD Trial in New Zealand; 35 families declined consent for future contact and 3 infants died after the neonatal period, leaving 1321 children eligible for follow-up (Figure). Of these, 1197 (91%) were assessed at 2 years. Three children were excluded because blood glucose concentration data were unavailable, leaving 1194 children in the analysis: 704 (59%) in the normoglycemia group and 490 (41%) in the hypoglycemia group (383 [78%] mild, 107 [22%] severe, 427 [87%] 1-2 episodes, and 63 [13%] recurrent episodes). Six hundred seven children (51%) had been randomized to prophylactic buccal dextrose gel and 587 (49%) to placebo gel.

**Figure.**
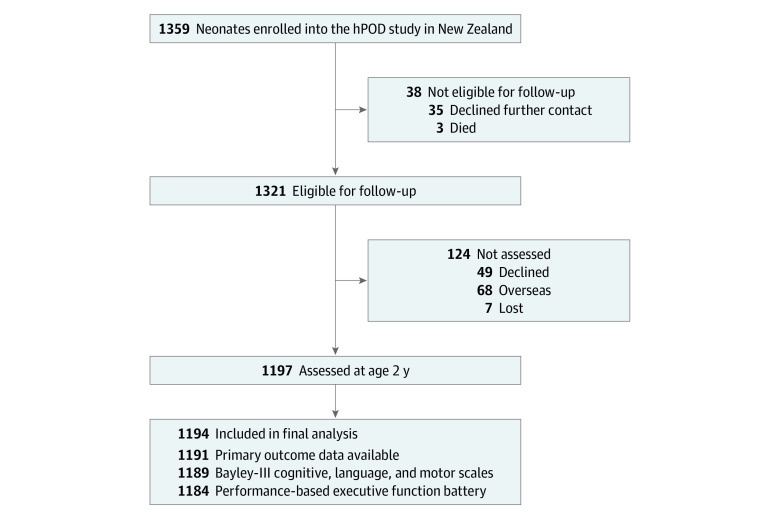
Study Flow Three of the 1197 children assessed who did not have blood glucose concentration data were excluded from the final analysis. Bayley-III indicates Bayley Scales of Infant and Toddler Development, Third Edition; hPOD, Hypoglycaemia Prevention With Oral Dextrose Trial.

Children were assessed at a mean (SD) corrected age of 24 (1.4) months; 578 (48%) were female and 616 (52%) were male. Maternal and child characteristics were similar between those assessed and not assessed, except more children of European mothers were assessed (eTable 1 in the Supplement). Among those assessed, children who became hypoglycemic were more likely to be male (270 [55%] vs 346 [49%]), have a lower gestational age (mean [SD], 37.8 [1.2] vs 38.2 [1.1] weeks) and birthweight (mean [SD], 3241 [639.3] vs 3403 [645.9] g), be a twin (51 [10%] vs 44 [6%]) or born preterm (63 [13%] vs 39 [6%]), admitted to NICU (75 [15%] vs 19 [3%]), and randomized to placebo gel (258 [43%] vs 229 [38%]) (Table 1). Primary outcome data were available for 1191 children: 487 in the hypoglycemia group and 704 in the normoglycemia group.

**Table 1.** Maternal and Neonatal Characteristics

Variable	No. (%)		
	Total	Hypoglycemia group	Normoglycemia group
**Mothers^a^**			
Total No.	1180	484	696
Age, mean (SD), y^b^	32.4 (5.3)	32.4 (5.3)	32.3 (5.3)
Prioritized ethnicity^c^			
Total No.	1179	484	695
Māori	213 (18.1)	74 (15.3)	139 (20.0)
Pacific	103 (8.7)	50 (10.3)	53 (7.6)
Asian	187 (15.9)	73 (15.1)	114 (16.4)
Indian	156 (13.2)	61 (12.6)	95 (13.7)
Other	61 (5.2)	24 (5.0)	37 (5.3)
European	459 (38.9)	202 (41.7)	257 (37.0)
Cesarean delivery	507 (43.0)	218 (45.0)	289 (41.5)
Diabetes	903 (76.5)	356 (73.6)	547 (78.6)
**Infants**			
Total No.	1194	490	704
Randomized to buccal dextrose gel	607 (50.8)	232 (47.3)	375 (53.3)
Female	578 (48.4)	220 (44.9)	358 (50.9)
Male	616 (51.6)	270 (55.1)	346 (49.1)
Gestation, mean (SD), wk^d^	38.0 (1.2)	37.8 (1.2)	38.2 (1.1)
Birthweight, mean (SD), g^d^	3337 (647.8)	3241 (639.3)	3403 (645.9)
Birthweight *z* score, mean (SD)	0.28 (1.2)	0.17 (1.2)	0.36 (1.2)
One of twins	95 (8.0)	51 (10.4)	44 (6.3)
Socioeconomic decile^e^			
No.	1189	487	702
1-2 (Least deprived)	179 (15.0)	70 (14.4)	109 (15.5)
3-4	228 (19.2)	90 (18.5)	138 (19.7)
5-6	252 (21.2)	114 (23.4)	138 (19.7)
7-8	279 (23.5)	117 (24.0)	162 (23.1)
9-10 (Most deprived)	251 (21.1)	96 (19.7)	155 (22.1)
Primary risk factor^f^			
Mother with diabetes	901 (75.5)	352 (71.8)	549 (78.0)
Preterm	102 (8.5)	63 (12.9)	39 (5.5)
Small	122 (10.2)	53 (10.8)	69 (9.8)
Large	69 (5.8)	22 (4.5)	47 (6.7)
First blood glucose concentration, mean (SD), mg/dL^g^	55.8 (13.2)	47.8 (12.7)	61.4 (10.3)
Hypoglycemia^h^	490 (41.0)	490 (100.0)	0
Mild^i^	383 (32.1)	383 (78.2)	0
Severe^j^	107 (9.0)	107 (21.8)	0
1-2 Episodes	427 (35.8)	427 (87.1)	0
Recurrent^k^	63 (5.3)	63 (12.9)	0
Treatment for hypoglycemia			
Buccal dextrose plus feed only	420 (35.2)	420 (85.7)	0
Intravenous dextrose	36 (3.0)	36 (7.3)	0
Admission to NICU	94 (7.9)	75 (15.3)	19 (2.7)

Abbreviation: NICU, neonatal intensive care unit.

SI conversion factor: To convert blood glucose to mmol/L, multiply by 0.0555.

^a^ Mothers included once per pregnancy because some mothers had 1 or more pregnancy and child in this cohort.

^b^ Missing data for 2 mothers (1 hypoglycemia, 1 normoglycemia).

^c^ Self-reported maternal ethnicity was prioritized using the New Zealand Ministry of Health classifications. Other included African, Canadian/Brazilian, Colombian, Fijian, Hawaiian, Indonesian, Kurdish, Latin American, Lebanese, Malaysian, Malaysian Indian, Mauritian, Middle Eastern, Nepalese, Somali, South African, and unspecified.

^d^ Missing data for 1 infant (normoglycemia).

^e^ New Zealand Socioeconomic Deprivation Index.

^f^ Primary risk factor was reported according to priority.

^g^ Missing data for 3 infants (normoglycemia).

^h^ Defined as 1 or more episodes of consecutive blood glucose concentrations less than 47 mg/dL.

^i^ Defined as 1 or more episodes of greater than or equal to 36 mg/dL and less than 47 mg/dL.

^j^ Defined as 1 or more episodes of less than 36 mg/dL.

^k^ Defined as 3 or more episodes.

### Primary Analyses

Compared with the normoglycemia group, children who experienced hypoglycemia were more likely to have neurosensory impairment (23% [111 of 487] vs 18% [125 of 704]; aRR, 1.28; 95% CI, 1.01-1.60) (Table 2). The risks of cognitive, language, or motor delay were similar between groups, but children who experienced hypoglycemia had lower Bayley-III cognitive (mean [SD], 97.8 [11.2] vs 99.0 [11.2]; aMD, −1.48 [95% CI, −2.79 to −0.18]) and motor (103.0 [10.3] vs 104.8 [10.8]; aMD, −2.05 [95% CI, −3.30 to −0.79]) composite scores (Table 2). There were no significant differences between groups in the risk of moderate or severe neurosensory impairment, cerebral palsy, developmental delay, Bayley-III language composite score, and executive function (Table 2; eTable 2 in the Supplement). In the hypoglycemia group, 2 children were blind and 3 had hearing impairment.

**Table 2.** Associations Between Neonatal Hypoglycemia and Neurosensory Impairment at Corrected Age 2 Years^a^

Primary outcome	No./total No. (%)	RD/MD (95% CI)	RR (95% CI)	Adjusted RR/MD (95% CI)^b^	*P* value
Normoglycemia	125/704 (17.8)				
Hypoglycemia^c^	111/487 (22.8)	5.04 (0.36 to 9.72)	1.28 (1.02 to 1.61)	1.28 (1.01 to 1.60)	.04
Severity of hypoglycemia^d^^,^^e^					.02
Mild	81/381 (21.3)	3.50 (−1.49 to 8.49)	1.20 (0.93 to 1.54)	1.18 (0.92 to 1.52)	.19
Severe	30/106 (28.3)	10.55 (1.51 to 19.59)	1.59 (1.13 to 2.25)	1.68 (1.20 to 2.36)	.003
Frequency of hypoglycemia ^f^^,^^g^					.08
1-2 episodes	99/424 (23.4)	5.59 (0.67 to 10.52)	1.32 (1.04 to 1.66)	1.29 (1.02 to 1.64)	.03
Recurrent	12/63 (19.1)	1.29 (−8.82 to 11.41)	1.07 (0.63 to 1.83)	1.06 (0.63 to 1.80)	.83

Abbreviations: MD, mean difference; RD, risk difference; RR, risk ratio.

^a^ Defined as any of the following: blindness (visual acuity <3/60 or >1.3 logMAR), hearing impairment requiring aids, cerebral palsy, developmental delay (Bayley-III cognitive, language, or motor composite score <85), or performance-based executive function total score more than 1.5 SD below the cohort mean.

^b^ Adjusted for study site, primary reason for risk of hypoglycemia, socioeconomic decile at birth, and multiple births.

^c^ An episode of hypoglycemia was defined as 1 or more episode of consecutive blood glucose concentrations less than 47 mg/dL (to convert glucose to millimoles per liter, multiply by 0.0555).

^d^ The severity of hypoglycemia was defined as none (all blood glucose concentrations ≥47 mg/dL), mild (≥1 episode of ≥36 and <47 mg/dL), and severe (≥1 episode of <36 mg/dL).

^e^ *P* values reported for the overall association between severity of hypoglycemia and outcome, none vs mild, and none vs severe.

^f^ The frequency of hypoglycemia was defined as none (all blood glucose concentrations ≥47 mg/dL), 1 to 2 episodes of consecutive blood glucose concentrations less than 47 mg/dL, and recurrent (≥3 episodes of consecutive blood glucose concentrations <47 mg/dL).

^g^ *P* values reported for the overall association between frequency of hypoglycemia and outcome, none vs 1 to 2 episodes, and none vs recurrent (ie, ≥3 episodes of consecutive blood glucose concentrations <47 mg/dL).

### Secondary Analyses

Compared with the normoglycemia group, children who experienced severe hypoglycemia had a higher risk of neurosensory impairment (28% [30 of 106] vs 18% [125 of 704]; aRR, 1.68 [95% CI, 1.20-2.36]). They also had a higher risk of developmental delay (24% [25 of 106] vs 16% [114 of 701]; aRR, 1.54 [95% CI, 1.05 to 2.26]), language delay (23% [24 of 106] vs 15% [104 of 702]; aRR, 1.65 [95% CI, 1.11 to 2.45]), motor delay (4% [4 of 106] vs 1% [10 of 702]; aRR, 3.40 [95% CI, 1.03 to 11.21]), and low performance on the snack delay task (85% [90 of 106] vs 73% [513 of 701]; aRR, 1.11 [95% CI, 1.01 to 1.22]) (Table 3; eTable 2 in the Supplement).

**Table 3.** Associations Between Neonatal Hypoglycemia and Neurocognitive Outcomes at Corrected Age 2 Years

Secondary outcome	No./total No. (%)		RD/MD (95% CI)	RR (95% CI)	Adjusted RR/MD (95% CI)^a^	*P* value^b^
	Hypoglycemia group (n = 490)^c^	Normoglycemia group (n = 704)^c^				
Moderate or severe neurosensory impairment^d^	20/487 (4.1)	20/704 (2.8)	1.27 (−0.88 to 3.42)	1.45 (0.79 to 2.66)	1.41 (0.76 to 2.62)	.28
Cerebral palsy	2/481 (0.4)	1/700 (0.1)	0.27 (−0.37 to 0.91)	NC	NC	
None	479/481 (99.6)	699/700 (99.9)	−0.27 (−0.91 to 0.37)	1 [Reference]	1 [Reference]	
Mild	0/481	1/700 (0.1)	−0.14 (NE)	NE	NE	
Moderate or severe	2/481 (0.4)	0/700	0.42 (NE)	NE	NE	
Developmental delay^e^	94/483 (19.5)	114/701 (16.3)	3.20 (−1.27 to 7.67)	1.20 (0.93 to 1.53)	1.18 (0.92 to 1.51)	.19
None	389/483 (80.5)	587/701 (83.7)	−3.20 (−7.67 to 1.27)	1 [Reference]	1 [Reference]	
Mild	79/483 (16.4)	94/701 (13.4)	2.95 (−1.21 to 7.10)	1.22 (0.93 to 1.61)	1.21 (0.92 to 1.59)	.18
Moderate or severe	15/483 (3.1)	20/701 (2.9)	0.25 (−1.73 to 2.23)	1.13 (0.58 to 2.18)	1.11 (0.57 to 2.17)	.76
Cognitive delay^e^	35/486 (7.2)	42/703 (6.0)	1.16 (−1.72 to 4.03)	1.21 (0.78 to 1.86)	1.22 (0.79 to 1.89)	.37
None	451/486 (92.8)	661/703 (94.0)	−1.23 (−4.12 to 1.67)	1 [Reference]	1 [Reference]	
Mild	31/486 (6.4)	36/703 (5.1)	1.26 (−1.46 to 3.98)	1.25 (0.78 to 1.99)	1.28 (0.80 to 2.06)	.30
Moderate or severe	4/486 (0.8)	6/703 (0.9)	−0.03 (−1.08 to 1.02)	0.98 (0.28 to 3.45)	0.82 (0.23 to 2.85)	.76
Language delay^e^	85/486 (17.5)	104/702 (14.8)	2.68 (−1.61 to 6.96)	1.18 (0.91 to 1.53)	1.16 (0.89 to 1.52)	.26
None	401/486 (82.5)	598/702 (85.2)	−2.68 (−6.96 to 1.61)	1 [Reference]	1 [Reference]	
Mild	71/486 (14.6)	87/702 (12.4)	2.22 (−1.76 to 6.20)	1.18 (0.89 to 1.58)	1.17 (0.87 to 1.57)	.29
Moderate or severe	14/486 (2.9)	17/702 (2.4)	0.46 (−1.42 to 2.33)	1.22 (0.61 to 2.45)	1.18 (0.58 to 2.40)	.64
Motor delay^e^	9/483 (1.9)	10/702 (1.4)	0.44 (−1.05 to 1.93)	1.31 (0.54 to 3.20)	1.38 (0.56 to 3.42)	.48
None	474/483 (98.1)	692/702 (98.6)	−0.44 (−1.93 to 1.05)	1 [Reference]	1 [Reference]	
Mild	8/483 (1.7)	7/702 (1.0)	0.66 (−0.70 to 2.02)	1.66 (0.60 to 4.54)	1.77 (0.63 to 4.96)	.28
Moderate or severe	1/483 (0.2)	3/702 (0.4)	−0.22 (−0.85 to 0.41)	0.49 (0.05 to 4.69)	0.53 (0.05 to 5.20)	.58
Bayley-III total scores, mean (SD) [total No.]^e^						
Cognitive score	97.8 (11.2) [486]	99.0 (11.2) [703]	−1.20 (−2.50 to 0.10)	NA	−1.48 (−2.79 to −0.18)	.03
Language score	98.3 (15.8) [486]	98.6 (15.0) [702]	−0.33 (−2.11 to 1.44)	NA	−0.51 (−2.29 to 1.28)	.58
Motor score	103.0 (10.3) [483]	104.8 (10.8) [702]	−1.82 (−3.05 to −0.59)	NA	−2.05 (−3.30 to −0.79)	.001
Poor executive function^f^	33/483 (6.8)	36/701 (5.1)	1.70 (−1.09 to 4.48)	1.33 (0.84 to 2.10)	1.31 (0.82 to 2.10)	.25
Executive function total score, mean (SD) [total No.]^g^	10.3 (4.5) [483]	10.6 (4.4) [701]	−0.30 (−0.82 to 0.22)	NA	−0.19 (−0.70 to 0.33)	.48

Abbreviations: Bayley-III, Bayley Scales of Infant and Toddler Development, Third Edition; MD, mean difference; NA, not applicable; NC, not calculated owing to small numbers; NE, not estimable; RD, risk difference; RR, risk ratio.

^a^ Adjusted for study site, primary reason for risk of hypoglycemia, socioeconomic decile at birth, and multiple births.

^b^ First *P* value reported in the outcome row is for the overall association between severity of hypoglycemia and outcome. The *P* values below in mild and severe rows are for the comparison between none vs mild or none vs severe.

^c^ An episode of hypoglycemia was defined as 1 or more episodes of consecutive blood glucose concentrations less than 47 mg/dL (to convert glucose to millimoles per liter, multiply by 0.0555).

^d^ Defined as any of the following: blindness, hearing impairment requiring aids, moderate or severe cerebral palsy (not walking yet or permanently nonambulant), or moderate or severe developmental delay.

^e^ One child in the hypoglycemia group was assigned a score of 49 for the Bayley-III cognitive, language, and motor scales because the child was unable to finish the assessment due to severe delay. Language, cognitive, and motor scales, mean (100), SD (15), and higher scores indicate better performance.

^f^ Defined as an executive function total score more than 1.5 SD below the mean.

^g^ Total score range 0 to 24 points; higher scores indicate better performance. eTable 2 in the Supplement presents the executive function component results.

Compared with Bayley-III motor composite scores in the normoglycemia group (mean [SD], 104.8 [10.8]), scores were lower in children who experienced mild (103.3 [9.9]; aMD, −1.69 [95% CI, −3.04 to −0.34]) and severe episodes (101.8 [11.3]; aMD, −3.39 [95% CI, −5.67 to −1.11]) (overall *P* = .003) (Table 4). Severity of hypoglycemia was not associated with moderate or severe neurosensory impairment, cerebral palsy, cognitive delay, or composite cognitive or language scores.

**Table 4.** Associations Between Severity of Neonatal Hypoglycemia and Neurocognitive Outcomes at Corrected Age 2 Years^a^

Variable	No./total (%)	RD/MD (95% CI)	RR (95% CI)	Adjusted RR/MD (95% CI)^b^	*P* value^c^
Moderate or severe neurosensory impairment^d^					.55
None	20/704 (2.8)	1 [Reference]	1 [Reference]	1 [Reference]	
Mild hypoglycemia	16/381 (4.2)	1.36 (−1.00 to 3.72)	1.48 (0.77 to 2.82)	1.42 (0.74 to 2.75)	.29
Severe hypoglycemia	4/106 (3.8)	0.93 (−2.90 to 4.77)	1.33 (0.46 to 3.82)	1.51 (0.51 to 4.52)	.46
Cerebral palsy					.41
None	1/700 (0.1)	1 [Reference]	1 [Reference]		
Mild hypoglycemia	1/377 (0.3)	0.12 (−0.47 to 0.71)	1.86 (0.12 to 29.69)	NC	
Severe hypoglycemia	1/104 (1.0)	0.82 (−1.08 to 2.72)	6.73 (0.42 to 107.23)	NC	
Developmental delay^e^					.11
None	114/701 (16.3)	1 [Reference]	1 [Reference]	1 [Reference]	
Mild hypoglycemia	69/377 (18.3)	2.04 (−2.73 to 6.81)	1.13 (0.86 to 1.48)	1.10 (0.83 to 1.45)	.51
Severe hypoglycemia	25/106 (23.6)	7.32 (−1.22 to 15.87)	1.45 (0.99 to 2.13)	1.54 (1.05 to 2.26)	.03
Cognitive delay^e^					.24
None	42/703 (6.0)	1 [Reference]	1 [Reference]	1 [Reference]	
Mild hypoglycemia	24/380 (6.3)	0.34 (−2.67 to 3.35)	1.06 (0.65 to 1.72)	1.07 (0.65 to 1.75)	.80
Severe hypoglycemia	11/106 (10.4)	4.40 (−1.67 to 10.48)	1.74 (0.92 to 3.27)	1.78 (0.94 to 3.37)	.08
Language delay^e^					.05
None	104/702 (14.8)	1 [Reference]	1 [Reference]	1 [Reference]	
Mild hypoglycemia	61/380 (16.1)	1.24 (−3.30 to 5.77)	1.08 (0.81 to 1.45)	1.06 (0.79 to 1.42)	.72
Severe hypoglycemia	24/106 (22.6)	7.83 (−0.58 to 16.23)	1.53 (1.03 to 2.27)	1.65 (1.11 to 2.45)	.01
Motor delay^e^					.13
None	10/702 (1.4)	1 [Reference]	1 [Reference]	1 [Reference]	
Mild hypoglycemia	5/377 (1.3)	−0.10 (−1.55 to 1.35)	0.93 (0.32 to 2.71)	1.02 (0.34 to 3.04)	.98
Severe hypoglycemia	4/106 (3.8)	2.35 (−1.39 to 6.09)	2.65 (0.84 to 8.31)	3.40 (1.03 to 11.21)	.04
Bayley-III cognitive score, mean (SD) [total]^e^					.08
None	99.0 (11.2) [703]	1 [Reference]	NA	1 [Reference]	
Mild hypoglycemia	97.9 (10.7) [380]	−1.11 (−2.51 to 0.30)	NA	−1.39 (−2.79 to −0.00)	.05
Severe hypoglycemia	97.5 (12.8) [106]	−1.54 (−3.84 to 0.76)	NA	−1.62 (−3.97 to 0.73)	.18
Bayley-III language score, mean (SD) [total]^e^					.49
None	98.6 (15.0) [702]	1 [Reference]	NA	1 [Reference]	
Mild hypoglycemia	98.6 (15.8) [380]	−0.02 (−1.94 to 1.90)	NA	−0.13 (−2.05 to 1.79)	.90
Severe hypoglycemia	97.2 (15.7) [106]	−1.45 (−4.59 to 1.68)	NA	−2.06 (−5.17 to 1.04)	.19
Bayley-III motor score, mean (SD) [total No.]^e^					.003
None	104.8 (10.8) [702]	1 [Reference]	NA	1 [Reference]	
Mild hypoglycemia	103.3 (9.9) [377]	−1.49 (−2.81 to −0.16)	NA	−1.69 (−3.04 to −0.34)	.01
Severe hypoglycemia	101.8 (11.3) [106]	−3.00 (−5.16 to −0.84)	NA	−3.39 (−5.67 to −1.11)	.004
Poor executive function^f^					.41
None	36/701 (5.1)	1 [Reference]	1 [Reference]	1 [Reference]	
Mild hypoglycemia	24/377 (6.4)	1.23 (−1.73 to 4.19)	1.24 (0.75 to 2.05)	1.24 (0.74 to 2.06)	.42
Severe hypoglycemia	9/106 (8.5)	3.36 (−2.21 to 8.92)	1.65 (0.82 to 3.34)	1.56 (0.75 to 3.24)	.23
Executive function total score, mean (SD) [total No.]^g^					.19
None	10.6 (4.4) [701]	1 [Reference]	NA	1 [Reference]	
Mild hypoglycemia	10.5 (4.5) [377]	−0.07 (−0.63 to 0.48)	NA	−0.01 (−0.54 to 0.57)	.96
Severe hypoglycemia	9.5 (4.5) [106]	−1.11 (−2.01 to −0.20)	NA	−0.82 (−1.73 to 0.10)	.08

Abbreviations: Bayley-III, Bayley Scales of Infant and Toddler Development, Third Edition; MD, mean difference; NA. not applicable; NC, not calculated owing to small numbers; RD, risk difference; RR, risk ratio.

^a^ The severity of hypoglycemia was defined as none (all blood glucose concentrations ≥47 mg/dL), mild (≥1 episode of ≥36 and <47 mg/dL), and severe (≥1 episode of <36 mg/dL) (to convert glucose to millimoles per liter, multiply by 0.0555).

^b^ Adjusted for study site, primary reason for risk of hypoglycemia, socioeconomic decile at birth, and multiple births.

^c^ *P* value reported is for the overall association between severity of hypoglycemia and outcome, none vs mild, and/or none vs severe.

^d^ Defined as any of the following: blindness, hearing impairment requiring aids, moderate or severe cerebral palsy (not walking yet or permanently nonambulant), or moderate or severe developmental delay.

^e^ One child in the hypoglycemia group was assigned a score of 49 for the Bayley-III cognitive, language, and motor scales because the child was unable to finish the assessment owing to severe delay. Language, cognitive, and motor scales, mean (100), SD (15), higher scores indicate better performance.

^f^ Poor executive function defined as an executive function total score more than 1.5 SD below the mean.

^g^ Total score range 0 to 24 points; higher scores indicate better performance. eTable 2 in the Supplement presents the executive function component results.

Compared with the normoglycemia group, children who experienced 1 to 2 episodes had a higher risk of neurosensory impairment (23% [99 of 424] vs 18% [125 of 704]; aRR, 1.29; 95% CI, 1.02-1.64) and lower Bayley-III cognitive (mean [SD] 97.5 [11.2] vs 99.0 [11.2]; aMD, −1.80; 95% CI, −3.17 to −0.44) and motor (102.8 [10.0] vs 104.8 [10.8]; aMD, −2.23; 95% CI, −3.53 to −0.92) composite scores (eTable 3 in the Supplement). However, children who experienced recurrent episodes did not have a higher risk of neurosensory impairment (19% [12 of 63]; aRR, 1.06; 95% CI, 0.63-1.80) (Table 2). The frequency of episodes was not associated with moderate or severe neurosensory impairment, cerebral palsy, developmental delay and components, or executive function.

In post hoc analysis restricted to children who experienced hypoglycemia, there was no interaction between frequency of episodes (1-2 vs recurrent) and treatment (buccal dextrose gel vs intravenous dextrose) with the risk of neurosensory impairment, moderate or severe neurosensory impairment, developmental delay and components, and executive function (eTable 4 in the Supplement).

In subgroup analyses, there was no interaction between hypoglycemia and primary reason for risk of hypoglycemia or infant sex with the risk of neurosensory impairment, moderate or severe neurosensory impairment, and their components (eTable 5 and eTable 6 in the Supplement). The findings were not altered after excluding 9 children with postneonatal diagnoses likely to affect the outcome (hypoglycemia group, 1% [3 of 490]; normoglycemia group, 1% [6 of 704]), or 202 children assessed outside the intended assessment window (hypoglycemia group, 16% [76 of 490]; normoglycemia group, 18% [126 of 704]) (eTable 7 and eTable 8 in the Supplement). After excluding 425 children whose first language was not English (hypoglycemia, 34% [166 of 490]; normoglycemia, 37% [259 of 704]), the effect size for the primary outcome was little changed but no longer statistically significant (18% [57 of 321] vs 14% [62 of 445]; aRR, 1.26; 95% CI, 0.90-1.76) (eTable 9 in the Supplement). After excluding 604 children randomized to prophylactic dextrose gel, the primary outcome findings were similar (47% [229 of 487] vs 53% [375 of 704]; aRR, 1.55; 95% CI, 1.10-2.18) (eTable 10 in the Supplement).

## Discussion

Children in this cohort study who experienced hypoglycemia were more likely than those who did not to have neurosensory impairment at corrected age 2 years, especially after severe but not recurrent episodes. Children who experienced any hypoglycemia had mean 1- to 2-point lower Bayley-III cognitive and motor scores, and children who experienced severe episodes had 3-point lower motor scores. Although these mean differences are small and unlikely to be of clinical relevance, the 5% absolute difference in overall neurosensory impairment is of more concern.

Our findings are consistent with previous reports that children who experienced neonatal hypoglycemia, especially severe episodes, were more likely to have poor outcomes, although this outcome has been reported only in later childhood, perhaps because affected skills, such as executive dysfunction and visual and motor impairment, are difficult to test at earlier ages.^7,18,19^ However, we found poorer neurodevelopmental outcomes at corrected age 2 years that were consistent between subgroup and sensitivity analyses.

One reason for this difference from previous reports may be confounding by neonatal comorbidities. Our cohort comprised well late preterm and full-term neonates without evidence of acute or imminent illness in the first hour after birth, of whom only 8% were later admitted to the NICU, making it easier to distinguish possible effects of hypoglycemia alone. Supporting this finding, to our knowledge, the only other study to detect neurosensory impairments in early childhood after hypoglycemia was a large (n = 101 060) population-based study that excluded many infants with other illnesses.^18^ In contrast, the Children With Hypoglycemia and Their Later Development Study reported similar risks of neurosensory impairment between children who did and did not experience hypoglycemia at both age 2 (n = 404) and 4.5 (n = 477) years.^7,20^ However, that cohort included children born moderately preterm, 4% had other neonatal illnesses as risk factors for hypoglycemia, and 50% had been admitted to the NICU.^7^

Another possible reason why previous studies have not detected neurodevelopmental impairment at young ages after hypoglycemia may have been limited power. We found relatively small differences between groups in most secondary outcomes (eg, 1-3 points in Bayley scores) and, in the post hoc analysis, for this cohort of 1194 children, the estimated power was 80% to detect a 7% absolute difference in neurosensory impairment between groups (2-tailed testing, α = .05). Most previous studies included far fewer infants. Furthermore, our inception cohort comprised participants in a randomized clinical trial that specified glucose testing using standard methods and prospective follow-up using standardized assessments.^14,15^ In contrast, some previous studies collected data retrospectively using hospital records, potentially increasing the risk of bias in assessment of exposures and outcomes.^9,19^

We found that even children who had experienced brief transitional hypoglycemia and were screened and treated with the intention of maintaining blood glucose concentrations greater than or equal to 47 mg/dL are at increased risk of neurodevelopmental impairment. One interpretation could be that current approaches to detection and treatment may ameliorate but not prevent later impairment. Continuous glucose monitoring has shown that neonates screened and treated as in this study spent a mean of 5 of the first 48 hours with interstitial glucose concentrations below the intended threshold.^20^ Furthermore, rapid correction of hypoglycemia and glucose instability after treatment, particularly with intravenous dextrose, have been associated with neurosensory impairment.^20,21^ The widely used treatment threshold of greater than or equal to 47 mg/dL also may not be optimal, although there is minimal reliable evidence about different treatment thresholds.^22^

It is also possible that hypoglycemia is a marker of neonates who are physiologically vulnerable and at risk of poor outcomes for other reasons.^23,24,25^ This possibility is consistent with the findings in this and other studies that the overall rate of neurodevelopmental impairment is high in children born at risk (19%-48%).^7,12,20,26,27^ Thus, treatment of hypoglycemia may prevent worsening of those outcomes but would not abolish this risk.

Our secondary analyses suggest that severity of hypoglycemia is more important than frequency for later neurodevelopment, although recurrent episodes were rare (5%), limiting the reliability of this finding. Neurosensory impairment has previously been reported in children who experienced frequent episodes.^7,28,29^ Because the number of episodes likely influences decisions around treatment and intravenous dextrose has been related to glycemic instability,^21^ we explored interactions between the frequency of hypoglycemia and treatment. Among children who experienced hypoglycemia, we found no evidence that neurocognitive outcomes differed between those treated with buccal dextrose gel or with intravenous dextrose, whether experiencing 1 to 2 or recurrent episodes. However, only 33 children were treated with intravenous dextrose, making interactions difficult to detect.

We found that executive function composite scores were similar between children who did and did not experience hypoglycemia, consistent with a previous report at age 2 years.^20^ However, in that cohort, hypoglycemia was associated with worse executive function at age 4.5 years.^7^ Because executive function is predictive of later academic outcomes^30^ and the overall rate of impairment is high, school-aged follow-up will be important in this and other cohorts.

### Strengths and Limitations

This study has strengths. The cohort was large and the follow-up rate was high at 91%. Data were collected prospectively and analyses were adjusted for known confounders. Because this was a cohort analysis of a randomized trial, there were consistent study entry criteria, excluding neonates with acute or imminent illnesses at 1 hour after birth.

This study also has limitations. First, the design was observational, so residual confounding was possible. Second, some findings may reflect type I errors because we did not adjust for multiplicity. Third, we did not use continuous glucose monitoring to more accurately detect the frequency of hypoglycemia.^31,32^ Fourth, although use of Bayley-III is reliable in detecting early developmental delays,^14^ it is less accurate to estimate later cognitive and motor functioning.^33,34,35^

## Conclusions

In this study, children born at risk but otherwise well who experienced neonatal hypoglycemia were more likely than those who did not to have neurosensory impairment at corrected age 2 years, with higher risks after severe episodes. Future studies should be large, consider confounding neonatal illnesses, and follow up children prospectively using individual assessments. Further research is also important to establish whether this association is causal and whether these early findings persist at school age.

## Notes

Supplement. **eTable 1.** Maternal and Infant Characteristics of Those Included and Not Included in This Analysis **eTable 2.** Associations Between Neonatal Hypoglycemia and Its Severity and Performance-Based Executive Function Tasks at 2 Years **eTable 3.** Associations Between Frequency of Neonatal Hypoglycemia and Neurocognitive Outcomes at 2 Years **eTable 4.** Interaction Between Treatment and the Relationship Between Frequency of Hypoglycemia and Neurocognitive Outcomes at 2 Years **eTable 5.** Selected Neurocognitive Outcomes in Children Who Did and Did Not Experience Hypoglycemia Who Had Different Primary Reasons for Risk of Hypoglycemia (Subgroup Analysis) **eTable 6.** Selected Neurocognitive Outcomes in Boys and Girls Who Did and Did Not Experience Hypoglycemia (Subgroup Analysis) **eTable 7.** Selected Neurocognitive Outcomes in Children Who Did and Did Not Experience Hypoglycemia, Excluding Children With Postneonatal Diagnoses Likely to Affect the Outcome (Sensitivity Analysis) **eTable 8.** Selected Neurocognitive Outcomes in Children Who Did and Did Not Experience Hypoglycemia, Excluding Children Assessed Outside of the Intended Assessment Window of 23 to 25 Months' Corrected Age (Sensitivity Analysis) **eTable 9.** Selected Neurocognitive Outcomes in Children Who Did and Did Not Experience Hypoglycemia, Excluding Children Whose First Language Was Not English (Sensitivity Analysis) **eTable 10.** The Primary Outcome of Neurosensory Impairment in Children Who Did and Did Not Experience Hypoglycemia, Excluding Children Randomized to Prophylactic Buccal Dextrose Gel (Sensitivity Analysis)
