# Supplementary material for: Neurocognitive Outcomes at Age 2 Years After Neonatal Hypoglycemia in a Cohort of Participants From the hPOD Randomized Trial
Source: JAMA Netw Open. 2022 Oct 11;5(10):e2235989. doi: 10.1001/jamanetworkopen.2022.35989 (PMC9554702; doi:10.1001/jamanetworkopen.2022.35989)
Supplement: Supplement. — eTable 1. Maternal and Infant Characteristics of Those Included and Not Included in This Analysis eTable 2. Associations Between Neonatal Hypoglycemia and Its Severity and Performance-Based Executive Function Tasks at 2 Years eTable 3. Associations Between Frequency of Neonatal Hypoglycemia and Neurocognitive Outcomes at 2 Years eTable 4. Interaction Between Treatment and the Relationship Between Frequency of Hypoglycemia and Neurocognitive Outcomes at 2 Years eTable 5. Selected Neurocognitive Outcomes in Children Who Did and Did Not Experience Hypoglycemia Who Had Different Primary Reasons for Risk of Hypoglycemia (Subgroup Analysis) eTable 6. Selected Neurocognitive Outcomes in Boys and Girls Who Did and Did Not Experience Hypoglycemia (Subgroup Analysis) eTable 7. Selected Neurocognitive Outcomes in Children Who Did and Did Not Experience Hypoglycemia, Excluding Children With Postneonatal Diagnoses Likely to Affect the Outcome (Sensitivity Analysis) eTable 8. Selected Neurocognitive Outcomes in Children Who Did and Did Not Experience Hypoglycemia, Excluding Children Assessed Outside of the Intended Assessment Window of 23 to 25 Months' Corrected Age (Sensitivity Analysis) eTable 9. Selected Neurocognitive Outcomes in Children Who Did and Did Not Experience Hypoglycemia, Excluding Children Whose First Language Was Not English (Sensitivity Analysis) eTable 10. The Primary Outcome of Neurosensory Impairment in Children Who Did and Did Not Experience Hypoglycemia, Excluding Children Randomized to Prophylactic Buccal Dextrose Gel (Sensitivity Analysis) [file jamanetwopen-e2235989-s001.pdf]

Supplementary Online Content

Edwards T, Alsweiler JM, Gamble GD, et al. Neurocognitive outcomes at age 2 years after neonatal hypoglycemia in a cohort of participants from the hPOD randomized trial. *JAMA Netw Open*. 2022;5(10):e2235989. doi:10.1001/jamanetworkopen.2022.35989

eTable 1. Maternal and Infant Characteristics of Those Included and Not Included in This Analysis

eTable 2. Associations Between Neonatal Hypoglycemia and Its Severity and Performance-Based Executive Function Tasks at 2 Years

eTable 3. Associations Between Frequency of Neonatal Hypoglycemia and Neurocognitive Outcomes at 2 Years

eTable 4. Interaction Between Treatment and the Association Between Frequency of Hypoglycemia and Neurocognitive Outcomes at 2 Years

eTable 5. Selected Neurocognitive Outcomes in Children Who Did and Did Not Experience Hypoglycemia Who Had Different Primary Reasons for Risk of Hypoglycemia (Subgroup Analysis)

eTable 6. Selected Neurocognitive Outcomes in Boys and Girls Who Did and Did Not Experience Hypoglycemia (Subgroup Analysis)

eTable 7. Selected Neurocognitive Outcomes in Children Who Did and Did Not Experience Hypoglycemia, Excluding Children With Postneonatal Diagnoses Likely to Affect the Outcome (Sensitivity Analysis)

eTable 8. Selected Neurocognitive Outcomes in Children Who Did and Did Not Experience Hypoglycemia, Excluding Children Assessed Outside of the Intended Assessment Window of 23 to 25 Months' Corrected Age (Sensitivity Analysis)

eTable 9. Selected Neurocognitive Outcomes in Children Who Did and Did Not Experience Hypoglycemia, Excluding Children Whose First Language Was Not English (Sensitivity Analysis)

eTable 10. The Primary Outcome of Neurosensory Impairment in Children Who Did and Did Not Experience Hypoglycemia, Excluding Children Randomized to Prophylactic Buccal Dextrose Gel (Sensitivity Analysis)

This supplementary material has been provided by the authors to give readers additional information about their work.

© 2022 Edwards T et al. *JAMA Network Open.*

eTable 1. Maternal and infant characteristics of those included and not included in this analysis

	Included<br>Total	Not included[a]<br>Total
**Mothers**[b]		
No.	1180	164
Age, mean (SD), years[c]	32.4 (5.3)	31.7 (6.0)
Prioritized ethnicity[d]	n = 1179	n = 155
Māori	213 (18.1)	34 (21.9)
Pacific	103 (8.7)	18 (11.6)
Asian	187 (15.9)	31 (20.0)
Indian	156 (13.2)	22 (14.2)
Other	61 (5.2)	5 (3.2)
European	459 (38.9)	45 (29.0)
Caesarean section	507 (43.0)	67 (41.4)
Mother with diabetes	903 (76.5)	118 (73.3)
**Infants**		
No.	1194	165
Randomized to buccal dextrose gel	607 (50.8)	74 (44.9)
Female	578 (48.4)	83 (50.3)
Gestation, mean (SD), weeks[e]	38.0 (1.2)	38.0 (1.2)
Birthweight, mean (SD), g[e]	3337 (647.8)	3276 (632.9)
Birthweight z-score, mean (SD)	0.28 (1.2)	0.17 (1.2)
One of Twins	95 (8.0)	9/163 (5.5)
Socioeconomic decile[f]	n = 1189	
Decile 1-2 (least deprived)	179 (15.0)	
Decile 3-4	228 (19.2)	
Decile 5-6	252 (21.2)	
Decile 7-8	279 (23.5)	
Decile 9-10 (most deprived)	251 (21.1)	
Primary risk factor[g]		
Infant of mother with diabetes	901 (75.5)	120 (72.7)
Preterm	102 (8.5)	14 (8.5)
Small	122 (10.2)	19 (11.5)

© 2022 Edwards T et al. *JAMA Network Open.*

	Included<br>Total	Not included[a]<br>Total
Large	69 (5.8)	12 (7.3)
First blood glucose concentration, mean (SD), mg/d[h]	55.8 (13.2)	55.5 (13.2)
Hypoglycemia[i]	490 (41.0)	59/157 (37.6)
Mild[j]	383 (32.1)	43/157 (27.4)
Severe[k]	107 (9.0)	16/157 (10.2)
1-2 episodes	427 (35.8)	52/157 (33.1)
Recurrent[l]	63 (5.3)	7/157 (4.5)
Treatment for hypoglycemia		
Buccal dextrose plus feed only	420 (35.2)	50/163 (30.7)
Intravenous dextrose	36 (3.0)	7/163 (4.3)
Admission to NICU	94 (7.9)	11 (6.7)

Data are number (%) or mean (SD). Abbreviations: SD, standard deviation; g, grams; mg/dL, milligrams per deciliter; NICU, neonatal intensive care unit.

SI conversion factor: To convert glucose to mmol/L, multiply by 0.0555.

[a] Includes 38 children not eligible for follow-up, 124 children not assessed, and three children assessed but with no blood glucose concentration data.

[b] Mothers included once per pregnancy because some mothers had ≥1 pregnancy and child in this cohort.

[c] Missing data for 2 included mother.

[d]Self-reported maternal ethnicity was prioritized using the New Zealand Ministry of Health classifications. "Other" included African, Canadian/Brazilian, Colombian, Fijian, Hawaiian, Indonesian, Kurdish, Latin American, Lebanese, Malaysian, Malaysian Indian, Mauritian, Middle Eastern, Nepalese, Somali, South African, and unspecified.

[e] Missing data for 1 included and 3 not included mothers.

[f] New Zealand Socioeconomic Deprivation Index (NZDep). This was not available for children not assessed.

[g] Primary risk factor was reported according to priority.

[h] Missing data for 3 included and 9 not included infants.

[i] Defined as ≥1 episode of consecutive blood glucose concentrations of <47 mg/dL.

[j] Defined as ≥1 episode of ≥36 and <47 mg/dL.

[k] Defined as ≥1 episode of <36 mg/dL.

[l] Defined as ≥3 episodes.

eTable 2. Associations between neonatal hypoglycemia and its severity and performance-based executive function tasks at 2 years

	No./total (%)	RD/MD (95% CI)	RR (95% CI)	Adjusted RR/MD[a] (95% CI)	*P* value[b]
Simple inhibition (snack delay task) score ≤2[c]					.02
None (reference)	513/701 (73.2)				
Any hypoglycemia	371/483 (76.8)	3.63 (-1.37 to 8.63)	1.05 (0.98 to 1.12)	1.03 (0.96 to 1.11)	.37
Mild hypoglycemia	281/377 (74.5)	1.36 (-4.14 to 6.85)	1.02 (0.95 to 1.10)	1.00 (0.93 to 1.08)	.98
Severe hypoglycemia	90/106 (84.9)	11.72 (4.15 to 19.30)	1.16 (1.06 to 1.27)	1.11 (1.01 to 1.22)	.02
Complex inhibition (fruit stroop task) score ≤2[c]					.41
None (reference)	351/698 (50.3)				
Any hypoglycemia	235/481 (48.9)	-1.43 (-7.24 to 4.38)	0.97 (0.86 to 1.09)	0.96 (0.86 to 1.08)	.53
Mild hypoglycemia	177/375 (47.2)	-3.09 (-9.36 to 3.19)	0.94 (0.82 to 1.07)	0.93 (0.81 to 1.06)	.26
Severe hypoglycemia	58/106 (54.7)	4.43 (-5.76 to 14.62)	1.09 (0.90 to 1.31)	1.08 (0.89 to 1.31)	.44
Complex inhibition (reverse categorisation task) score ≤2[c]					.92
None (reference)	544/699 (77.8)				
Any hypoglycemia	372/481 (77.3)	-0.49 (-5.34 to 4.36)	0.99 (0.93 to 1.06)	0.99 (0.93 to 1.05)	.70
Mild hypoglycemia	286/375 (76.3)	-1.56 (-6.86 to 3.74)	0.98 (0.91 to 1.05)	0.99 (0.92 to 1.06)	.72
Severe hypoglycemia	86/106 (81.1)	3.31 (-4.77 to 11.38)	1.04 (0.94 to 1.15)	0.99 (0.90 to 1.09)	.89
Attentional flexibility (multisearch/multilocation task) score ≤2[c]					.49
None (reference)	114/699 (16.3)				
Hypoglycemia	80/481 (16.6)	0.32 (-3.99 to 4.64)	1.02 (0.79 to 1.32)	0.93 (0.71 to 1.22)	.61
Mild hypoglycemia	58/375 (15.5)	-0.84 (-5.42 to 3.73)	0.95 (0.71 to 1.27)	0.87 (0.64 to 1.16)	.34
Severe hypoglycemia	22/106 (20.8)	4.45 (-3.76 to 12.65)	1.27 (0.85 to 1.92)	1.09 (0.71 to 1.68)	.70

Abbreviations: RD, risk difference; MD, mean difference; CI, confidence interval; RR, risk ratio. An episode of hypoglycemia was defined as ≥1 episode of consecutive blood glucose concentrations of <47 mg/dL. The severity of hypoglycemia was defined as none (all blood glucose concentrations ≥47 mg/dL), mild (≥1 episode of ≥36 and <47 mg/dL), and severe (≥1 episode of <36 mg/dL).

[a] Models compare relative risks of none vs any episode of hypoglycemia, none vs mild hypoglycemia, and none vs severe hypoglycemia. Adjustments are for study site, primary reason for risk of hypoglycemia, socioeconomic decile at birth and multiple births.

[b] First *P* value reported in the outcome row is for the overall association between severity of hypoglycemia and outcome. *P* values below in the any, mild and severe rows are for the comparison between none vs any or none vs mild or none vs severe.

[c] Low performance defined as a score of ≤2, range 0-6, higher scores indicate better performance.

© 2022 Edwards T et al. *JAMA Network Open.*

eTable 3. Associations between frequency of neonatal hypoglycemia[a] and neurocognitive outcomes at 2 years

	No./total (%)	RD/MD (95% CI)	RR (95% CI)	Adjusted RR/MD[b] (95% CI)	*P* value[c]
Moderate or severe neurosensory impairment[d]					.53
None (reference)	20/704 (2.8)				
1-2 episodes of hypoglycemia	17/424 (4.0)	1.17 (-1.07 to 3.41)	1.41 (0.75 to 2.67)	1.36 (0.71 to 2.61)	.36
≥3 episodes of hypoglycemia	3/63 (4.8)	1.92 (-3.49 to 7.33)	1.68 (0.51 to 5.50)	1.63 (0.49 to 5.35)	.42
Cerebral palsy					.21
None (reference)	1/700 (0.1)				
1-2 episodes of hypoglycemia	1/419 (0.2)	0.10 (-0.45 to 0.64)	1.67 (0.10 to 26.72)	NC	.84
≥3 episodes of hypoglycemia	1/62 (1.6)	1.47 (-1.68 to 4.62)	11.29 (0.71 to 179.10)	NC	.10
Developmental delay[e]					.12
None (reference)	114/701 (16.3)				
1-2 episodes of hypoglycemia	86/420 (20.5)	4.21 (-0.52 to 8.95)	1.26 (0.98 to 1.62)	1.24 (0.96 to 1.61)	.09
≥3 episodes of hypoglycemia	8/63 (12.7)	-3.56 (-12.24 to 5.11)	0.78 (0.40 to 1.53)	0.75 (0.39 to 1.46)	.40
Cognitive delay[e]					.28
None (reference)	42/703 (6.0)				
1-2 episodes of hypoglycemia	33/423 (7.8)	1.83 (-1.28 to 4.93)	1.31 (0.84 to 2.03)	1.33 (0.85 to 2.08)	.21
≥3 episodes of hypoglycemia	2/63 (3.2)	-2.80 (-7.48 to 1.88)	0.53 (0.13 to 2.15)	0.56 (0.14 to 2.25)	.41

© 2022 Edwards T et al. *JAMA Network Open.*

	No./total (%)	RD/MD (95% CI)	RR (95% CI)	Adjusted RR/MD[b] (95% CI)	*P* value[c]
Language delay[e]					.09
None (reference)	104/702 (14.8)				
1-2 episodes of hypoglycemia	79/423 (18.7)	3.43 (-1.14 to 8.01)	1.26 (0.97 to 1.65)	1.25 (0.96 to 1.64)	.10
≥3 episodes of hypoglycemia	6/63 (9.5)	-5.29 (-13.01 to 2.43)	0.64 (0.29 to 1.41)	0.61 (0.28 to 1.34)	.22
Motor delay[e]					.45
None (reference)	10/702 (1.4)				
1-2 episodes of hypoglycemia	7/420 (1.7)	0.24 (-1.27 to 1.75)	1.17 (0.45 to 3.05)	1.20 (0.45 to 3.17)	.71
≥3 episodes of hypoglycemia	2/63 (3.2)	1.75 (-2.67 to 6.17)	2.23 (0.50 to 9.97)	2.42 (0.54 to 10.86)	.25
Bayley-III cognitive score, mean (SD) [total][e]					.01
None (reference)	99.0 (11.2) [n = 703]				
1-2 episodes of hypoglycemia	97.5 (11.2) [n = 423]	-1.57 (-2.93 to -0.22)		-1.80 (-3.17 to -0.44)	.01
≥3 episodes of hypoglycemia	100.3 (11.3) [n = 63]	1.28 (-1.62 to 4.17		0.81 (-2.04 to 3.66)	.52
Bayley-III language score, mean (SD) [total][e]					.04
None (reference)	98.6 (15.0) [n = 702]				
1-2 episodes of hypoglycemia	97.6 (15.5) [n = 423]	-1.01 (-2.86 to 0.84)		-1.18 (-3.03 to 0.67)	.21

© 2022 Edwards T et al. *JAMA Network Open.*

	No./total (%)	RD/MD (95% CI)	RR (95% CI)	Adjusted RR/MD[b] (95% CI)	*P* value[c]
≥3 episodes of hypoglycemia	102.8 (16.9) [n = 63]	4.21 (0.26 to 8.16)		3.79 (-0.05 to 7.62)	.05
Bayley-III motor score, mean (SD) [total][e]					.004
None (reference)	104.8 (10.8) [n = 702]				
1-2 episodes of hypoglycemia	102.8 (10.0) [n = 420]	-2.01 (-3.29 to -0.73)		-2.23 (-3.53 to -0.92)	.001
≥3 episodes of hypoglycemia	104.2 (12.0) [n = 63]	-0.56 (-3.29 to 2.18)		-0.77 (-3.59 to 2.05)	.59
Poor executive function[f]					.41
None (reference)	36/701 (5.1)				
1-2 episodes of hypoglycemia	30/420 (7.1)	2.01 (-0.95 to 4.97)	1.39 (0.87 to 2.22)	1.35 (0.83 to 2.18)	.22
≥3 episodes of hypoglycemia	3/63 (4.8)	-0.37 (-5.89 to 5.14)	0.93 (0.29 to 2.93)	0.99 (0.31 to 3.12)	.98
Executive function total score, mean (SD) [total] [g]					.71
None (reference)	10.6 (4.4) [n = 701]				
1-2 episodes of hypoglycemia	10.2 (4.4) [n = 420]	-0.34 (-0.88 to 0.20)		-0.21 (-0.74 to 0.33)	.45
≥3 episodes of hypoglycemia	10.6 (4.8) [n = 63]	-0.03 (-1.18 to 1.12)		0.01 (-1.12 to 1.14)	.99
Simple inhibition (snack delay task) score ≤2[h]					.16
None (reference)	513/701 (73.2)				

	No./total (%)	RD/MD (95% CI)	RR (95% CI)	Adjusted RR/MD[b] (95% CI)	*P* value[c]
1-2 episodes of hypoglycemia	327/420 (77.9)	4.68 (-0.48 to 9.83)	1.06 (0.99 to 1.14)	1.05 (0.98 to 1.13)	.19
≥3 episodes of hypoglycemia	44/63 (69.8)	-3.34 (-15.16 to 8.48)	0.95 (0.81 to 1.13)	0.90 (0.76 to 1.07)	.24
Complex inhibition (fruit stroop task) score ≤2[h]					.70
None (reference)	351/698 (50.3)				
1-2 episodes of hypoglycemia	207/418 (49.5)	-0.77 (-6.83 to 5.30)	0.98 (0.87 to 1.11)	0.97 (0.86 to 1.10)	.62
≥3 episodes of hypoglycemia	28/63 (44.4)	-5.84 (-18.68 to 7.00)	0.88 (0.66 to 1.18)	0.90 (0.67 to 1.19)	.46
Complex inhibition (reverse categorisation task) score ≤2[h]					.92
None (reference)	544/699 (77.8)				
1-2 episodes of hypoglycemia	324/419 (77.3)	-0.50 (-5.56 to 4.56)	0.99 (0.93 to 1.06)	0.99 (0.93 to 1.06)	.76
≥3 episodes of hypoglycemia	48/62 (77.4)	-0.41 (-11.28 to 10.46)	0.99 (0.86 to 1.14)	1.01 (0.88 to 1.16)	.88
Attentional flexibility (multisearch/multilocation task) score ≤2[h]					.88
None (reference)	114/699 (16.3)				
1-2 episodes of hypoglycemia	70/418 (16.8)	0.44 (-4.08 to 4.95)	1.03 (0.78 to 1.35)	0.92 (0.69 to 1.21)	.54
≥3 episodes of hypoglycemia	10/63 (15.9)	-0.44 (-9.88 to 9.01)	0.97 (0.54 to 1.76)	0.88 (0.49 to 1.60)	.68

Abbreviations: RD, risk difference; MD, mean difference; CI, confidence interval; RR, risk ratio; NC, not calculated due to small numbers; Bayley-III, Bayley Scales of Infant and Toddler Development, Third Edition (mean=100, SD=15, range=40-160, higher scores indicate better performance); SD, standard deviation.

[a] The frequency of hypoglycemia was defined as none (all blood glucose concentrations ≥47 mg/dL), 1-2 episodes of consecutive blood glucose concentrations of <47 mg/dL, and recurrent (≥3 episodes of consecutive blood glucose concentrations of <47 mg/dL).

[b] Adjusted for study site, primary reason for risk of hypoglycemia, socioeconomic decile at birth and multiple births.

© 2022 Edwards T et al. *JAMA Network Open.*

[c] First *P* value reported in the outcome row is for the overall association between frequency of hypoglycemia and outcome. *P* values below in 1-2 and ≥3 rows are for the comparison between none vs 1-2 episodes or none vs ≥3 episodes.

[d] Defined as any of: blindness, hearing impairment requiring aids, moderate or severe cerebral palsy (not walking yet or permanently non-ambulant) or moderate or severe developmental delay.

[e] One child in the hypoglycemia group was assigned a score of 49 for the Bayley-III cognitive, language and motor scales because the child was unable to finish the assessment due to severe delay. Language, cognitive and motor scales, mean 100, SD 15, higher scores indicate better performance.

[f] Defined as an executive function total score more than 1.5 SD below the mean.

[g] Total score range 0-24 points, higher scores indicate better performance.

[h] Low performance defined as a score of ≤2, range 0-6, higher scores indicate better performance.

© 2022 Edwards T et al. *JAMA Network Open.*

eTable 4. Interaction between treatment and the association between frequency of hypoglycemia and neurocognitive outcomes at 2 years

	No./total (%)					
	≥3 episodes (N = 63)	1-2 episodes (N = 427)	RD/MD (95% CI)	RR (95% CI)	Adjusted RR/MD[a] (95% CI)	*P* value for interaction
**Neurosensory impairment**						
Buccal dextrose gel plus feed	9/51 (17.6)	85/360 (23.6)	-5.96 (-17.34 to 5.42)	0.75 (0.40 to 1.39)	0.75 (0.41 to 1.38)	.78
Intravenous dextrose	3/12 (25.0)	4/21 (19.0)	5.95 (-24.96 to 36.86)	1.31 (0.33 to 5.17)	0.90 (0.00 to 366.29)	
**Moderate or severe neurosensory impairment**						
Buccal dextrose gel plus feed	1/51 (2.0)	15/360 (4.2)	-2.21 (-6.55 to 2.14)	0.47 (0.06 to 3.51)	0.56 (0.08 to 4.11)	.39
Intravenous dextrose	2/12 (16.7)	1/21 (4.8)	11.90 (-12.00 to 35.81)	3.50 (0.32 to 38.06)	1.31 (0.02 to 84.85)	
**Developmental delay[b]**						
Buccal dextrose gel plus feed	6/51 (11.8)	72/356 (20.2)	-8.46 (-18.27 to 1.35)	0.58 (0.27 to 1.27)	0.57 (0.26 to 1.23)	.84
Intravenous dextrose	2/12 (16.7)	4/21 (19.1)	-2.38 (-30.43 to 25.67)	0.88 (0.18 to 4.35)	0.64 (0.01 to 31.81)	
**Cognitive delay[b]**						
Buccal dextrose gel plus feed	1/51 (2.0)	28/359 (7.8)	-5.84 (-10.56 to -1.12)	0.25 (0.03 to 1.82)	0.30 (0.04 to 2.16)	.78
Intravenous dextrose	1/12 (8.3)	2/21 (9.5)	-1.19 (-22.06 to 19.68)	0.88 (0.08 to 9.51)	1.04 (0.01 to 123.21)	
**Language delay[b]**						
Buccal dextrose gel plus feed	4/51 (7.8)	67/359 (18.7)	-10.82 (-19.25 to -2.39)	0.42 (0.16 to 1.11)	0.40 (0.15 to 1.05)	.85
Intravenous dextrose	2/12 (16.7)	4/21 (19.1)	-2.38 (-30.43 to 25.67)	0.88 (0.18 to 4.35)	0.64 (0.01 to 31.81)	
**Motor delay[b]**						
Buccal dextrose gel plus feed	1/51 (2.0)	6/356 (1.7)	0.28 (-3.77 to 4.32)	1.16 (0.14 to 9.53)	1.20 (0.15 to 9.61)	.92
Intravenous dextrose	1/12 (8.3)	1/21 (4.8)	3.57 (-15.26 to 22.40)	1.75 (0.11 to 28.44)	1.13 (0.01 to 97.55)	
**Bayley-III cognitive score, mean (SD)[b]**						
Buccal dextrose gel plus feed	101.6 (11.5) [n = 51]	97.5 (11.0) [n = 359]	4.11 (0.86 to 7.36)		4.18 (0.90 to 7.45)	.23
Intravenous dextrose	95.0 (9.3) [n = 12]	98.1 (16.2) [n = 21]	-3.10 (-13.55 to 7.36)		1.43 (-11.03 to 13.88)	

© 2022 Edwards T et al. *JAMA Network Open.*

	≥3 episodes	1-2 episodes	RD/MD (95% CI)	RR (95% CI)	Adjusted RR/MD[a] (95% CI)	*P* value for interaction
**Bayley-III language score, mean (SD)[b]**						
Buccal dextrose gel plus feed	105.5 (16.9) [n = 51]	97.6 (15.7) [n = 359]	7.89 (3.23 to 12.56)		7.80 (3.13 to 12.48)	.07
Intravenous dextrose	91.6 (11.5) [n = 12]	97.3 (11.5) [n = 21]	-5.75 (-16.52 to 5.02)		-2.92 (-17.43 to 11.59)	
**Bayley-III motor score, mean (SD)[b]**						
Buccal dextrose gel plus feed	105.4 (12.3) [n = 51]	102.9 (10.2) [n = 356]	2.49 (-0.58 to 5.57)		2.72 (-0.39 to 5.84)	.36
Intravenous dextrose	99.5 (9.7) [n = 12]	100.8 (9.2) [n = 21]	-1.26 (-8.19 to 5.67)		-1.73 (-12.25 to 8.78)	
**Poor executive function[c]**						
Buccal dextrose gel plus feed	2/51 (3.9)	27/356 (7.6)	-3.66 (-9.68 to 2.35)	0.52 (0.13 to 2.12)	0.42 (0.10 to 1.73)	.52
Intravenous dextrose	1/12 (8.3)	1/21 (4.8)	3.57 (-15.26 to 22.40)	1.75 (0.11 to 28.44)	1.13 (0.01 to 97.55)	
**Executive function total score, mean (SD)[d]**						
Buccal dextrose gel plus feed	10.6 (4.8) [n = 51]	10.3 (4.5) [n = 356]	0.29 (-1.05 to 1.64)		0.47 (-0.88 to 1.82)	.50
Intravenous dextrose	10.5 (5.1) [n = 12]	9.0 (3.5) [n = 21]	1.45 (-1.58 to 4.48)		0.75 (-3.31 to 4.80)	

Abbreviations: RD, risk difference; CI, confidence interval; RR, risk ratio; Bayley-III, Bayley Scales of Infant and Toddler Development, Third Edition (mean=100, SD=15, range=40-160, higher scores indicate better performance); SD, standard deviation.

An episode of hypoglycemia was defined as ≥1 episode of consecutive blood glucose concentrations of <47 mg/dL.

Nine children who received treatment but did not have recorded episodes of hypoglycemia were excluded.

[a] Generalized linear models adjusted for study site, primary reason for risk of hypoglycemia, socioeconomic decile at birth and multiple births.

[b] One child in the hypoglycemia group was assigned a score of 49 for the Bayley-III cognitive, language and motor scales because the child was unable to finish the assessment due to severe delay.

[c] Poor executive function was defined as an executive function total score more than 1.5 SD below the mean.

[d] Total score range 0-24 points, higher scores indicate better performance.

© 2022 Edwards T et al. *JAMA Network Open.*

eTable 5. Selected neurocognitive outcomes in children who did and did not experience hypoglycemia who had different primary reasons for risk of hypoglycemia (subgroup analysis)

Outcome	Subgroup	No./total (%)					
		Hypoglycemia[a] (n = 490)	No hypoglycemia (n = 704)	RD/MD (95% CI)	RR (95% CI)	Adjusted RR/MD[b] (95% CI)	*P* value for interaction
Neurosensory impairment	IDM	87/349 (24.9)	97/549 (17.7)	7.26 (1.71 to 12.82)	1.41 (1.09 to 1.82)	1.42 (1.10 to 1.83)	.18
	Other	24/138 (17.4)	28/155 (18.1)	-0.67 (-9.47 to 8.12)	0.96 (0.59 to 1.58)	1.00 (0.61 to 1.63)	
Moderate or severe neurosensory impairment	IDM	16/349 (4.6)	14/549 (2.6)	2.03 (-0.53 to 4.60)	1.80 (0.89 to 3.64)	1.81 (0.88 to 3.71)	.26
	Other	4/138 (2.9)	6/155 (3.9)	-0.97 (-5.12 to 3.18)	0.75 (0.21 to 2.61)	0.74 (0.20 to 2.77)	
Blindness	IDM	1/349 (0.3)	0/548 (0.0)	0.29 (NE)	NE	NE	
	Other	1/138 (0.7)	0/154 (0.0)	0.72 (NE)	NE	NE	
Hearing impairment	IDM	2/349 (0.6)	0/548 (0.0)	0.57 (NE)	NE	NE	
	Other	1/138 (0.7)	0/154 (0.0)	0.72 (NE)	NE	NE	
Cerebral palsy	IDM	2/344 (0.6)	0/547 (0.0)	0.58 (NE)	NE	NE	
	Other	0/137 (0.0)	1/153 (0.7)	-0.65 (NE)	NE	NE	
Developmental delay[c]	IDM	75/346 (21.7)	88/548 (16.1)	5.62 (0.29 to 10.94)	1.35 (1.02 to 1.78)	1.34 (1.02 to 1.77)	.12
	Other	19/137 (13.9)	26/153 (17.0)	-3.13 (-11.46 to 5.21)	0.82 (0.47 to 1.41)	0.84 (0.50 to 1.44)	
Cognitive delay[c]	IDM	28/348 (8.1)	32/549 (5.8)	2.22 (-1.25 to 5.69)	1.38 (0.85 to 2.25)	1.42 (0.87 to 2.34)	.29
	Other	7/138 (5.1)	10/154 (6.5)	-1.42 (-6.79 to 3.95)	0.78 (0.30 to 2.00)	1.02 (0.39 to 2.72)	
Language delay[c]	IDM	67/348 (19.3)	80/548 (14.6)	4.65 (-0.44 to 9.75)	1.32 (0.98 to 1.77)	1.31 (0.97 to 1.76)	.18
	Other	18/138 (13.0)	24/154 (15.6)	-2.54 (-10.60 to 5.52)	0.84 (0.47 to 1.48)	0.97 (0.84 to 1.12)	
Motor delay[c]	IDM	6/346 (1.7)	7/549 (1.3)	0.46 (-1.21 to 2.13)	1.36 (0.46 to 4.02)	1.53 (0.51 to 4.62)	.90
	Other	3/137 (2.2)	3/153 (2.0)	0.23 (-3.08 to 3.53)	1.12 (0.23 to 5.48)	1.43 (0.27 to 7.62)	

© 2022 Edwards T et al. *JAMA Network Open.*

**Outcome**	**Subgroup**	**Hypoglycemia[a] (n = 490)**	**No hypoglycemia (n = 704)**	**RD/MD (95% CI)**	**RR (95% CI)**	**Adjusted RR/MD[b] (95% CI)**	***P* value for interaction**
Bayley-III cognitive score, mean (SD)[c]	IDM	97.3 (11.1) [n = 348]	99.0 (10.9) [n = 549]	-1.69 (-3.16 to -0.21)		-1.79 (-3.26 to -0.32)	.24
	Other	99.2 (11.5) [n = 138]	99.2 (12.4) [n = 154]	-0.02 (-2.79 to 2.75)		-0.63 (-3.42 to 2.17)	
Bayley-III language score, mean (SD)[c]	IDM	97.3 (15.7) [n = 348]	98.5 (14.7) [n = 548]	-1.21 (-3.24 to 0.82)		-1.13 (-3.14 to 0.89)	.13
	Other	100.9 (15.8) [n = 138]	99.2 (16.1) [n = 154]	1.71 (-1.97 to 5.39)		1.17 (-2.56 to 4.89)	
Bayley-III motor score, mean (SD)[c]	IDM	102.4 (10.1) [n = 346]	104.7 (10.5) [n = 549]	-2.35 (-3.74 to -0.95)		-2.35 (-3.76 to -0.93)	.24
	Other	104.5 (10.4) [n = 137]	105.1 (12.0) [n = 153]	-0.57 (-3.18 to 2.04)		-1.19 (-3.84 to 1.46)	
Poor executive function[d]	IDM	24/345 (7.0)	29/548 (5.3)	1.67 (-1.61 to 4.94)	1.31 (0.78 to 2.22)	1.38 (0.81 to 2.35)	.99
	Other	9/138 (6.5)	7/153 (4.6)	1.95 (-3.36 to 7.25)	1.43 (0.54 to 3.74)	1.10 (0.41 to 2.95)	
Executive function total score, mean (SD)[e]	IDM	10.2 (4.4) [n = 345]	10.5 (4.4) [n = 548]	-0.37 (-0.97 to 0.22)		-0.32 (-0.91 to 0.28)	.61
	Other	10.6 (4.6) [n = 138]	10.8 (4.5) [n = 153]	-0.19 (-1.25 to 0.86)		-0.06 (-1.12 to 1.00)	
Simple inhibition (snack delay task) score ≤2	IDM	274/345 (79.4)	407/548 (74.3)	5.15 (-0.48 to 10.78)	1.07 (0.99 to 1.15)	1.04 (0.96 to 1.13)	.46
	Other	97/138 (70.3)	106/153 (69.3)	1.01 (-9.60 to 11.62)	1.01 (0.87 to 1.18)	0.96 (0.81 to 1.14)	
Complex inhibition (fruit stroop task) score ≤2	IDM	175/343 (51.0)	282/547 (51.6)	-0.53 (-7.29 to 6.22)	0.99 (0.87 to 1.13)	1.00 (0.88 to 1.14)	.70
	Other	60/138 (43.5)	69/151 (45.7)	-2.22 (-13.73 to 9.30)	0.95 (0.73 to 1.23)	0.95 (0.73 to 1.23)	
Complex inhibition (reverse categorisation task) score ≤2	IDM	272/344 (79.1)	423/547 (77.3)	1.74 (-3.82 to 7.30)	1.02 (0.95 to 1.10)	1.02 (0.95 to 1.09)	.21
	Other	100/137 (73.0)	121/152 (79.6)	-6.61 (-16.47 to 3.24)	0.92 (0.80 to 1.04)	0.97 (0.86 to 1.10)	

© 2022 Edwards T et al. *JAMA Network Open.*

Outcome	Subgroup	Hypoglycemia[a] (n = 490)	No hypoglycemia (n = 704)	RD/MD (95% CI)	RR (95% CI)	Adjusted RR/MD[b] (95% CI)	*P* value for interaction
Attentional flexibility (multisearch/ multilocation task) score ≤2	IDM	56/345 (16.2)	87/548 (15.9)	0.36 (-4.60 to 5.31)	1.02 (0.75 to 1.39)	1.00 (0.73 to 1.36)	.73
	Other	24/136 (17.7)	27/151 (17.9)	0.23 (-9.13 to 8.66)	0.99 (0.60 to 1.63)	0.87 (0.53 to 1.44)	

Abbreviations: RD, risk difference; CI, confidence interval; RR, risk ratio; NE, not estimable; Bayley-III, Bayley-Scales of Infant and Toddler Development, Third Edition (mean=100, SD=15, range=40-160, higher scores indicate better performance); SD, standard deviation.

[a] An episode of hypoglycemia was defined as ≥1 episode of consecutive blood glucose concentrations of <47 mg/dL.

[b] Generalized linear models adjusted for study site, primary reason for risk of hypoglycemia, socioeconomic decile at birth and multiple births.

[c] One child who became hypoglycaemic was assigned a score of 49 for the Bayley-III cognitive, language and motor scales due to severe delay.

[d] Poor executive function defined as an executive function total score more than 1.5 SD below the mean.

[e] Total score range 0-24 points, higher scores indicate better performance.

© 2022 Edwards T et al. *JAMA Network Open.*

eTable 6. Selected neurocognitive outcomes in boys and girls who did and did not experience hypoglycemia (subgroup analysis)

Outcome	Subgroup	Hypoglycemia[a] (n = 490)	No hypoglycemia (n = 704)	RD/MD (95% CI)	RR (95% CI)	Adjusted RR/MD[b] (95% CI)	*P* value for interaction
Neurosensory impairment	Boys	76/268 (28.4)	76/346 (22.0)	6.39 (-0.56 to 13.35)	1.29 (0.98 to 1.70)	1.27 (0.95 to 1.69)	.85
	Girls	35/219 (16.0)	49/358 (13.7)	2.30 (-3.74 to 8.33)	1.17 (0.78 to 1.74)	1.12 (0.75 to 1.67)	
Moderate or severe neurosensory impairment	Boys	15/268 (5.6)	13/346 (3.8)	1.84 (-1.57 to 5.25)	1.49 (0.72 to 3.08)	1.60 (0.76 to 3.38)	.70
	Girls	5/219 (2.3)	7/358 (2.0)	0.33 (-2.12 to 2.78)	1.17 (0.37 to 3.64)	0.98 (0.32 to 3.06)	
Blindness	Boys	1/268 (0.4)	0/346 (0.0)	0.37 (NE)	NE	NE	
	Girls	1/219 (0.5)	0/356 (0.0)	0.46 (NE)	NE	NE	
Hearing impairment	Boys	2/268 (0.8)	0/346 (0.0)	0.75 (NE)	NE	NE	
	Girls	1/219 (0.5)	0/356 (0.0)	0.46 (NE)	NE	NE	
Cerebral palsy	Boys	0/264 (0.0)	1/345 (0.3)	-0.29 (NE)	NE	NE	
	Girls	2/217 (0.9)	0/355 (0.0)	0.92 (NE)	NE	NE	
Developmental delay[c]	Boys	65/266 (24.4)	72/345 (20.9)	3.57 (-3.16 to 10.29)	1.17 (0.87 to 1.57)	1.12 (0.83 to 1.53)	.90
	Girls	29/217 (13.4)	42/356 (11.8)	1.57 (-4.08 to 7.21)	1.13 (0.73 to 1.76)	1.06 (0.68 to 1.65)	
Cognitive delay[c]	Boys	28/268 (10.5)	31/345 (9.0)	1.46 (-3.29 to 6.22)	1.16 (0.71 to 1.89)	1.13 (0.68 to 1.86)	.93
	Girls	7/218 (3.2)	11/358 (3.1)	0.14 (-2.81 to 3.09)	1.05 (0.41 to 2.66)	1.11 (0.43 to 2.84)	
Language delay[c]	Boys	59/268 (22.0)	66/345 (19.1)	2.88 (-3.60 to 9.37)	1.15 (0.84 to 1.57)	1.12 (0.81 to 1.55)	.98
	Girls	26/218 (11.9)	38/357 (10.6)	1.28 (-4.09 to 6.66)	1.12 (0.70 to 1.79)	1.03 (0.65 to 1.66)	
Motor delay[c]	Boys	7/266 (2.6)	8/345 (2.3)	0.31 (-2.19 to 2.81)	1.13 (0.42 to 3.10)	1.29 (0.46 to 3.64)	.88
	Girls	2/217 (0.9)	2/357 (0.6)	0.36 (-1.13 to 1.85)	1.65 (0.23 to 11.64)	1.38 (0.19 to 10.16)	

© 2022 Edwards T et al. *JAMA Network Open.*

Outcome	Subgroup	Hypoglycemia[a] (n = 490)	No hypoglycemia (n = 704)	RD/MD (95% CI)	RR (95% CI)	Adjusted RR/MD[b] (95% CI)	*P* value for interaction
Bayley-III cognitive score, mean (SD)[c]	Boys	96.3 (12.2) [n = 268]	97.7 (12.0) [n = 345]	-1.37 (-3.31 to 0.56)		-1.72 (-3.68 to 0.25)	.63
	Girls	99.7 (9.5) [n = 218]	100.3 (10.4) [n = 358]	-0.63 (-2.33 to 1.06)		-0.87 (-2.56 to 0.83)	
Bayley-III language score, mean (SD)[c]	Boys	95.1 (16.0) [n = 268]	96.3 (14.9) [n = 345]	-1.15 (-3.60 to 1.31)		-1.68 (-4.18 to 0.82)	.19
	Girls	102.2 (14.7) [n = 218]	100.9 (14.9) [n = 357]	1.29 (-1.21 to 3.78)		1.36 (-1.13 to 3.85)	
Bayley-III motor score, mean (SD)[c]	Boys	101.7 (10.4) [n = 266]	103.6 (11.3) [n = 345]	-1.96 (-3.71 to -0.21)		-2.45 (-4.25 to -0.65)	.63
	Girls	104.6 (9.9) [n = 217]	105.9 (10.2) [n = 357]	-1.35 (-3.06 to 0.35)		-1.58 (-3.32 to 0.16)	
Poor executive function[d]	Boys	22/266 (9.0)	16/344 (4.7)	3.62 (-0.38 to 7.62)	1.94 (1.05 to 3.58)	1.76 (0.93 to 3.35)	.09
	Girls	9/217 (4.2)	20/357 (5.6)	-1.46 (-5.03 to 2.12)	0.74 (0.34 to 1.60)	0.77 (0.36 to 1.67)	
Executive function total score, mean (SD)[e]	Boys	9.8 (4.5) [n = 266]	10.3 (4.5) [n = 344]	-0.56 (-1.28 to 0.15)		-0.38 (-1.11 to 0.35)	.35
	Girls	10.9 (4.4) [n = 217]	10.8 (4.4) [n = 357]	0.09 (-0.65 to 0.83)		0.18 (-0.56 to 0.93)	
Simple inhibition (snack delay task) score ≤2	Boys	210/266 (79.0)	263/344 (76.5)	2.49 (-4.16 to 9.15)	1.03 (0.95 to 1.12)	0.99 (0.89 to 1.10)	.22
	Girls	161/217 (74.2)	250/357 (70.0)	4.17 (-3.37 to 11.70)	1.06 (0.95 to 1.18)	1.05 (0.95 to 1.17)	
Complex inhibition (fruit stroop task) score ≤2	Boys	140/264 (53.0)	169/342 (49.4)	3.62 (-4.42 to 11.65)	1.07 (0.92 to 1.25)	1.06 (0.90 to 1.24)	.10
	Girls	95/217 (43.8)	182/356 (51.1)	-7.35 (-15.76 to 1.07)	0.86 (0.71 to 1.03)	0.84 (0.70 to 1.01)	
Complex inhibition (reverse categorisation task) score ≤2	Boys	205/265 (77.4)	272/343 (79.3)	-1.94 (-8.57 to 4.69)	0.98 (0.90 to 1.06)	0.97 (0.88 to 1.07)	.45
	Girls	167/216 (77.3)	272/356 (76.4)	0.91 (-6.22 to 8.04)	1.01 (0.92 to 1.11)	1.03 (0.94 to 1.13)	
Attentional flexibility (multisearch/multilocation task) score ≤2	Boys	48/265 (18.1)	55/343 (16.0)	2.08 (-3.98 to 8.14)	1.13 (0.79 to 1.61)	0.97 (0.66 to 1.41)	.64
	Girls	32/216 (14.8)	59/356 (16.6)	-1.76 (-7.88 to 4.37)	0.89 (0.60 to 1.33)	0.85 (0.57 to 1.27)	

© 2022 Edwards T et al. *JAMA Network Open.*

Abbreviations: RD, risk difference; CI, confidence interval; RR, risk ratio; NE, not estimable; Bayley-III, Bayley-Scales of Infant and Toddler Development, Third Edition (mean=100, SD=15, range=40-160, higher scores indicate better performance); SD, standard deviation.

[a] An episode of hypoglycemia was defined as ≥1 episode of consecutive blood glucose concentrations of <47 mg/dL.

[b] Generalized linear models adjusted for study site, primary reason for risk of hypoglycemia, socioeconomic decile at birth and multiple births.

[c] One child who became hypoglycaemic was assigned a score of 49 for the Bayley-III cognitive, language and motor scales due to severe delay.

[d] Poor executive function defined as an executive function total score more than 1.5 SD below the mean.

[e] Total score range 0-24 points, higher scores indicate better performance.

© 2022 Edwards T et al. *JAMA Network Open.*

eTable 7. Selected neurocognitive outcomes in children who did and did not experience hypoglycemia, excluding children with postneonatal diagnoses likely to affect the outcome (sensitivity analysis)

	Analysis group	No./total (%)					
		Hypoglycemia[a] (N = 490)	No hypoglycemia (n = 704)	RD/MD (95% CI)	RR (95% CI)	Adjusted RR/MD[b] (95% CI)	*P* value
Neurosensory impairment	Whole cohort	111/487 (22.8)	125/704 (17.8)	5.04 (0.36 to 9.72)	1.28 (1.02 to 1.61)	1.28 (1.01 to 1.60)	.04
	Diagnoses excluded	110/484 (22.7)	123/698 (17.6)	5.11 (0.42 to 9.79)	1.29 (1.02 to 1.62)	1.29 (1.02 to 1.62)	.03
Moderate or severe neurosensory impairment	Whole cohort	20/487 (4.1)	20/704 (2.8)	1.27 (-0.88 to 3.42)	1.45 (0.79 to 2.66)	1.41 (0.76 to 2.62)	.28
	Diagnoses excluded	19/484 (3.9)	18/698 (2.6)	1.35 (-0.75 to 3.44)	1.52 (0.81 to 2.87)	1.53 (0.80 to 2.92)	.20
Cerebral palsy	Whole cohort	2/481 (0.4)	1/700 (0.1)	0.27 (-0.37 to 0.91)	2.91 (0.26 to 32.09)	2.44 (0.20 to 29.14)	.48
	Diagnoses excluded	2/478 (0.4)	1/694 (0.1)	0.27 (-0.37 to 0.92)	2.90 (0.26 to 32.01)	2.44 (0.20 to 29.14)	.48
Developmental delay[c]	Whole cohort	94/483 (19.5)	114/701 (16.3)	3.20 (-1.27 to 7.67)	1.20 (0.93 to 1.53)	1.18 (0.92 to 1.51)	.19
	Diagnoses excluded	93/480 (19.4)	112/695 (16.1)	3.26 (-1.21 to 7.73)	1.20 (0.94 to 1.54)	1.19 (0.93 to 1.53)	.17
Cognitive delay[c]	Whole cohort	35/486 (7.2)	42/703 (6.0)	1.16 (-1.72 to 4.03)	1.21 (0.78 to 1.86)	1.22 (0.79 to 1.89)	.37
	Diagnoses excluded	34/483 (7.0)	41/697 (5.9)	1.16 (-1.72 to 4.03)	1.20 (0.77 to 1.86)	1.23 (0.79 to 1.92)	.37
Language delay[c]	Whole cohort	85/486 (17.5)	104/702 (14.8)	2.68 (-1.61 to 6.96)	1.18 (0.91 to 1.53)	1.16 (0.89 to 1.52)	.26
	Diagnoses excluded	84/483 (17.4)	102/696 (14.7)	2.74 (-1.55 to 7.02)	1.19 (0.91 to 1.55)	1.18 (0.90 to 1.54)	.23
Motor delay[c]	Whole cohort	9/483 (1.9)	10/702 (1.4)	0.44 (-1.05 to 1.93)	1.31 (0.54 to 3.20)	1.38 (0.56 to 3.42)	.48
	Diagnoses excluded	8/480 (1.7)	8/696 (1.2)	0.52 (-0.88 to 1.91)	1.45 (0.55 to 3.84)	1.61 (0.60 to 4.34)	.35
Bayley-III cognitive score, mean (SD)[c]	Whole cohort	97.8 (11.2) [n = 486]	99.0 (11.2) [n = 703]	-1.20 (-2.50 to 0.10)		-1.48 (-2.79 to -0.18)	.03
	Diagnoses excluded	97.9 (11.1) [n = 483]	99.1 (11.1) [n = 697]	-1.20 (-2.49 to 0.10)		-1.50 (-2.80 to -0.21)	.02

© 2022 Edwards T et al. *JAMA Network Open.*

	Analysis group	Hypoglycemia[a] (N = 490)	No hypoglycemia (n = 704)	RD/MD (95% CI)	RR (95% CI)	Adjusted RR/MD[b] (95% CI)	*P* value
Bayley-III language score, mean (SD)[c]	Whole cohort	98.3 (15.8) [n = 486]	98.6 (15.0) [n = 702]	-0.33 (-2.11 to 1.44)		-0.51 (-2.29 to 1.28)	.58
	Diagnoses excluded	98.3 (15.7) [n = 483]	98.7 (14.9) [n = 696]	-0.36 (-2.13 to 1.42)		-0.56 (-2.34 to 1.22)	.54
Bayley-III motor score, mean (SD)[c]	Whole cohort	103.0 (10.3) [n = 483]	104.8 (10.8) [n = 702]	-1.82 (-3.05 to -0.59)		-2.05 (-3.30 to -0.79)	.001
	Diagnoses excluded	103.0 (10.2) [n = 480]	104.9 (10.6) [n = 696]	-1.88 (-3.09 to -0.67)		-2.14 (-3.38 to -0.90)	.001
Poor executive function[d]	Whole cohort	33/483 (6.8)	36/701 (5.1)	1.70 (-1.09 to 4.48)	1.33 (0.84 to 2.10)	1.31 (0.82 to 2.10)	.25
	Diagnoses excluded	32/480 (6.7)	34/695 (4.9)	1.78 (-0.98 to 4.53)	1.36 (0.85 to 2.18)	1.37 (0.85 to 2.21)	.20
Executive function total score, mean (SD)[e]	Whole cohort	10.3 (4.5) [n = 483]	10.6 (4.4) [n = 701]	-0.30 (-0.82 to 0.22)		-0.19 (-0.70 to 0.33)	.48
	Diagnoses excluded	10.3 (4.5) [n = 480]	10.6 (4.4) [n = 695]	-0.31 (-0.83 to 0.21)		-0.20 (-0.72 to 0.32)	.45

Abbreviations: RD, risk difference; CI, confidence interval; RR, risk ratio; Bayley-III, Bayley-Scales of Infant and Toddler Development, Third Edition (mean=100, SD=15, range=40-160, higher scores indicate better performance); SD, standard deviation.

Excluded 9 children with a post-neonatal diagnoses likely to affect the outcome including: 3 in the hypoglycemia group (1 mengitis, 1 near drowning, 1 genetic condition) and 6 in the normoglycemia group (3 meningitis, 1 congenital abnormality, 2 genetic conditions).

[a] An episode of hypoglycemia was defined as ≥1 episode of consecutive blood glucose concentrations of <47 mg/dL.

[b] Generalized linear models adjusted for study site, primary reason for risk of hypoglycemia, socioeconomic decile at birth and multiple births.

[c] One child who became hypoglycaemic was assigned a score of 49 for the Bayley-III cognitive, language and motor scales due to severe delay.

[d] Poor executive function defined as an executive function total score more than 1.5 SD below the mean.

[e] Composite score range 0-24 points, higher scores indicate better performance.

© 2022 Edwards T et al. *JAMA Network Open.*

eTable 8. Selected neurocognitive outcomes in children who did and did not experience hypoglycemia, excluding children assessed outside of the intended assessment window of 23 to 25 months' corrected age (sensitivity analysis)

Outcome	Analysis group	No./total (%)					
		Hypoglycemia[a] (N = 490)	No hypoglycemia (n = 704)	RD/MD (95% CI)	RR (95% CI)	Adjusted RR/MD[b] (95% CI)	*P* value
Neurosensory impairment	Whole cohort	111/487 (22.8)	125/704 (17.8)	5.04 (0.36 to 9.72)	1.28 (1.02 to 1.61)	1.28 (1.01 to 1.60)	.04
	Within window	92/411 (22.4)	98/578 (17.0)	5.43 (0.36 to 10.49)	1.32 (1.02 to 1.70)	1.32 (1.02 to 1.71)	.04
Moderate or severe neurosensory impairment	Whole cohort	20/487 (4.1)	20/704 (2.8)	1.27 (-0.88 to 3.42)	1.45 (0.79 to 2.66)	1.41 (0.76 to 2.62)	.28
	Within window	15/411 (3.7)	15/578 (2.6)	1.05 (-1.18 to 3.29)	1.41 (0.69 to 2.85)	1.27 (0.61 to 2.61)	.52
Cerebral palsy	Whole cohort	2/481 (0.4)	1/700 (0.1)	0.27 (-0.37 to 0.91)	2.91 (0.26 to 32.09)	2.44 (0.20 to 29.14)	.48
	Within window	2/408 (0.5)	1/576 (0.2)	0.32 (-0.44 to 1.08)	2.82 (0.26 to 31.13)	2.41 (0.20 to 28.37)	.49
Developmental delay[c]	Whole cohort	94/483 (19.5)	114/701 (16.3)	3.20 (-1.27 to 7.67)	1.20 (0.93 to 1.53)	1.18 (0.92 to 1.51)	.19
	Within window	79/410 (19.3)	90/576 (15.6)	3.64 (-1.20 to 8.48)	1.23 (0.94 to 1.62)	1.23 (0.93 to 1.62)	.15
Cognitive delay[c]	Whole cohort	35/486 (7.2)	42/703 (6.0)	1.16 (-1.72 to 4.03)	1.21 (0.78 to 1.86)	1.22 (0.79 to 1.89)	.37
	Within window	30/410 (7.3)	32/577 (5.6)	1.77 (-1.37 to 4.91)	1.32 (0.81 to 2.14)	1.32 (0.81 to 2.16)	.27
Language delay[c]	Whole cohort	85/486 (17.5)	104/702 (14.8)	2.68 (-1.61 to 6.96)	1.18 (0.91 to 1.53)	1.16 (0.89 to 1.52)	.26
	Within window	72/410 (17.6)	83/576 (14.4)	3.15 (-1.52 to 7.83)	1.22 (0.91 to 1.63)	1.22 (0.91 to 1.64)	.19
Motor delay[c]	Whole cohort	9/483 (1.9)	10/702 (1.4)	0.44 (-1.05 to 1.93)	1.31 (0.54 to 3.20)	1.38 (0.56 to 3.42)	.48
	Within window	7/410 (1.7)	7/577 (1.2)	0.49 (-1.05 to 2.04)	1.41 (0.50 to 3.99)	1.43 (0.49 to 4.14)	.51
Bayley-III cognitive score, mean (SD)[c]	Whole cohort	97.8 (11.2) [n = 486]	99.0 (11.2) [n = 703]	-1.20 (-2.50 to 0.10)		-1.48 (-2.79 to -0.18)	.03
	Within window	98.3 (11.5) [n = 410]	99.4 (11.1) [n = 577]	-1.12 (-2.55 to 0.31)		-1.36 (-2.81 to 0.08)	.06
Bayley-III language score, mean (SD)[c]	Whole cohort	98.3 (15.8) [n = 486]	98.6 (15.0) [n = 702]	-0.33 (-2.11 to 1.44)		-0.51 (-2.29 to 1.28)	.58
	Within window	98.8 (15.9) [n = 410]	98.9 (15.2) [n = 576]	-0.04 (-2.00 to 1.93)		-0.25 (-2.24 to 1.74)	.81
Bayley-III motor score, mean (SD)[c]	Whole cohort	103.0 (10.3) [n = 483]	104.8 (10.8) [n = 702]	-1.82 (-3.05 to -0.59)		-2.05 (-3.30 to -0.79)	.001
	Within window	103.2 (10.0) [n = 410]	104.9 (10.4) [n = 577]	-1.77 (-3.07 to -0.47)		-1.95 (-3.29 to -0.62)	.004

© 2022 Edwards T et al. *JAMA Network Open.*

Outcome	Analysis group	Hypoglycemia[a] (N = 490)	No hypoglycemia (n = 704)	RD/MD (95% CI)	RR (95% CI)	Adjusted RR/MD[b] (95% CI)	*P* value
Poor executive function[d]	Whole cohort	33/483 (6.8)	36/701 (5.1)	1.70 (-1.09 to 4.48)	1.33 (0.84 to 2.10)	1.31 (0.82 to 2.10)	.25
	Within window	27/408 (6.6)	30/576 (5.2)	1.41 (-1.61 to 4.43)	1.27 (0.77 to 2.11)	1.25 (0.75 to 2.11)	.39
Executive function total score, mean (SD)[e]	Whole cohort	10.3 (4.5) [n = 483]	10.6 (4.4) [n = 701]	-0.30 (-0.82 to 0.22)		-0.19 (-0.70 to 0.33)	.48
	Within window	10.2 (4.4) [n = 408]	10.4 (4.3) [n = 576]	-0.16 (-0.71 to 0.39)		-0.05 (-0.60 to 0.51)	.87

Abbreviations: RD, risk difference; CI, confidence interval; RR, risk ratio; Bayley-III, Bayley-Scales of Infant and Toddler Development, Third Edition (mean=100, SD=15, range=40-160, higher scores indicate better performance); SD, standard deviation.

Within window = excluded 202 children (76 in the hypoglycemia group and 126 in the normoglycemia group) assessed outside the intended study window period.

[a] An episode of hypoglycemia was defined as ≥1 episode of consecutive blood glucose concentrations of <47 mg/dL.

[b] Generalized linear models adjusted for study site, primary reason for risk of hypoglycemia, socioeconomic decile at birth and multiple births.

[c] One child who became hypoglycaemic was assigned a score of 49 for the Bayley-III cognitive, language and motor scales due to severe delay.

[d] Poor executive function defined as an executive function total score more than 1.5 SD below the mean.

[e] Composite score range 0-24 points, higher scores indicate better performance.

© 2022 Edwards T et al. *JAMA Network Open.*

eTable 9. Selected neurocognitive outcomes in children who did and did not experience hypoglycemia, excluding children whose first language was not English (sensitivity analysis)

Outcome	Analysis group	Hypoglycemia[a] (N = 490)	No hypoglycemia (n = 704)	RD/MD (95% CI)	RR (95% CI)	Adjusted RR/MD[b] (95% CI)	*P* value
Neurosensory impairment	Whole cohort	111/487 (22.8)	125/704 (17.8)	5.04 (0.36 to 9.72)	1.28 (1.02 to 1.61)	1.28 (1.01 to 1.60)	.04
	First language English	57/321 (17.8)	62/445 (13.9)	3.82 (-1.46 to 9.11)	1.27 (0.92 to 1.77)	1.26 (0.90 to 1.76)	.18
Moderate or severe neurosensory impairment	Whole cohort	20/487 (4.1)	20/704 (2.8)	1.27 (-0.88 to 3.42)	1.45 (0.79 to 2.66)	1.41 (0.76 to 2.62)	.28
	First language English	12/321 (3.7)	12/445 (2.7)	1.04 (-1.53 to 3.61)	1.39 (0.63 to 3.05)	1.17 (0.53 to 2.60)	.69
Cerebral palsy	Whole cohort	2/481 (0.4)	1/700 (0.1)	0.27 (-0.37 to 0.91)	2.91 (0.26 to 32.09)	2.44 (0.20 to 29.14)	.48
	First language English	2/316 (0.6)	1/443 (0.2)	0.41 (-0.57 to 1.39)	2.80 (0.25 to 30.90)	2.40 (0.21 to 26.92)	.48
Developmental delay[c]	Whole cohort	94/483 (19.5)	114/701 (16.3)	3.20 (-1.27 to 7.67)	1.20 (0.93 to 1.53)	1.18 (0.92 to 1.51)	.19
	First language English	45/319 (14.1)	57/442 (12.9)	1.21 (-3.73 to 6.15)	1.09 (0.76 to 1.57)	1.04 (0.72 to 1.50)	.85
Cognitive delay[c]	Whole cohort	35/486 (7.2)	42/703 (6.0)	1.16 (-1.72 to 4.03)	1.21 (0.78 to 1.86)	1.22 (0.79 to 1.89)	.37
	First language English	17/320 (5.3)	23/444 (5.2)	0.13 (-3.08 to 3.35)	1.03 (0.56 to 1.89)	0.91 (0.49 to 1.69)	.77
Language delay[c]	Whole cohort	85/486 (17.5)	104/702 (14.8)	2.68 (-1.61 to 6.96)	1.18 (0.91 to 1.53)	1.16 (0.89 to 1.52)	.26
	First language English	40/320 (12.5)	52/443 (11.7)	0.76 (-3.95 to 5.47)	1.06 (0.72 to 1.57)	1.00 (0.68 to 1.49)	.98
Motor delay[c]	Whole cohort	9/483 (1.9)	10/702 (1.4)	0.44 (-1.05 to 1.93)	1.31 (0.54 to 3.20)	1.38 (0.56 to 3.42)	.48
	First language English	4/319 (1.3)	7/443 (1.6)	-0.33 (-2.01 to 1.36)	0.79 (0.23 to 2.69)	0.80 (0.23 to 2.75)	.72
Bayley-III cognitive score, mean (SD)[c]	Whole cohort	97.8 (11.2) [n = 486]	99.0 (11.2) [n = 703]	-1.20 (-2.50 to 0.10)		-1.48 (-2.79 to -0.18)	.03
	First language English	99.2 (11.1) [n = 320]	100.3 (11.5) [n = 444]	-1.05 (-2.68 to 0.58)		-1.36 (-2.99 to 0.28)	.10
Bayley-III language score, mean (SD)[c]	Whole cohort	98.3 (15.8) [n = 486]	98.6 (15.0) [n = 702]	-0.33 (-2.11 to 1.44)		-0.51 (-2.29 to 1.28)	.58
	First language English	101.3 (15.9) [n = 324]	101.2 (15.5) [n = 443]	0.07 (-2.18 to 2.33)		-0.16 (-2.42 to 2.11)	.89

© 2022 Edwards T et al. *JAMA Network Open.*

Outcome	Analysis group	Hypoglycemia[a] (N = 490)	No hypoglycemia (n = 704)	RD/MD (95% CI)	RR (95% CI)	Adjusted RR/MD[b] (95% CI)	*P* value
Bayley-III motor score, mean (SD)[c]	Whole cohort	103.0 (10.3) [n = 483]	104.8 (10.8) [n = 702]	-1.82 (-3.05 to -0.59)		-2.05 (-3.30 to -0.79)	.001
	First language English	103.7 (10.0) [n = 319]	105.2 (10.9) [n = 443]	-1.54 (-3.06 to -0.02)		-1.86 (-3.42 to -0.30)	.02
Poor executive function[d]	Whole cohort	33/483 (6.8)	36/701 (5.1)	1.70 (-1.09 to 4.48)	1.33 (0.84 to 2.10)	1.31 (0.82 to 2.10)	.25
	First language English	15/318 (4.7)	17/443 (3.8)	0.88 (-0.02 to 3.82)	1.23 (0.62 to 2.43)	1.28 (0.64 to 2.55)	.49
Executive function total score, mean (SD)[e]	Whole cohort	10.3 (4.5) [n = 483]	10.6 (4.4) [n = 701]	-0.30 (-0.82 to 0.22)		-0.19 (-0.70 to 0.33)	.48
	First language English	10.8 (4.3) [n = 318]	10.9 (4.4) [n = 443]	-0.06 (-0.69 to 0.57)		0.00 (-0.64 to 0.64)	1.00
Complex inhibition (fruit stroop task) score ≤2	Whole cohort	235/481 (48.9)	351/698 (50.3)	-1.43 (-7.24 to 4.38)	0.97 (0.86 to 1.09)	0.96 (0.86 to 1.08)	.53
	First language English	148/317 (46.7)	212/440 (48.2)	-1.49 (-8.71 to 5.73)	0.97 (0.83 to 1.13)	0.96 (0.82 to 1.11)	.57
Complex inhibition (reverse categorisation task) score ≤2	Whole cohort	372/481 (77.3)	544/699 (77.8)	-0.49 (-5.34 to 4.36)	0.99 (0.93 to 1.06)	0.99 (0.93 to 1.05)	.70
	First language English	238/317 (75.1)	331/441 (75.1)	0.02 (-6.23 to 6.28)	1.00 (0.92 to 1.09)	0.97 (0.89 to 1.06)	.56

Abbreviations: RD, risk difference; CI, confidence interval; RR, risk ratio; Bayley-III, Bayley-Scales of Infant and Toddler Development, Third Edition (mean=100, SD=15, range=40-160, higher scores indicate better performance); SD, standard deviation.

First language English = excluded 425 children (166 in the hypoglycemia group and 259 in the normoglycemia group).

[a] An episode of hypoglycemia was defined as ≥1 episode of consecutive blood glucose concentrations of <47 mg/dL.

[b] Generalized linear models adjusted for study site, primary reason for risk of hypoglycemia, socioeconomic decile at birth and multiple births.

[c] One child who became hypoglycaemic was assigned a score of 49 for the Bayley-III cognitive, language and motor scales due to severe delay.

[d] Poor executive function defined as an executive function total score more than 1.5 SD below the mean.

[e] Composite score range 0-24 points, higher scores indicate better performance.

© 2022 Edwards T et al. *JAMA Network Open.*

eTable 10. The primary outcome of neurosensory impairment in children who did and did not experience hypoglycemia, excluding children randomized to prophylactic buccal dextrose gel (sensitivity analysis)

	No./total (%)	RD (95% CI)	RR (95% CI)	Adjusted RR[a] (95% CI)	*P* value
Normoglycemia (reference)[b]	49/329 (14.9)				
Hypoglycemia[c]	61/258 (23.6)	8.75 (2.28 to 15.22)	1.59 (1.13 to 2.23)	1.55 (1.10 to 2.18)	.01
Mild hypoglycemia[d]	42/204 (20.6)	5.70 (-1.07 to 12.46)	1.38 (0.95 to 2.01)	1.34 (0.92 to 1.97)	.13
Severe hypoglycemia[e]	19/54 (35.2)	20.29 (6.94 to 33.64)	2.36 (1.51 to 3.69)	2.39 (1.49 to 3.84)	<.001
1-2 episodes of hypoglycemia	53/224 (23.7)	8.77 (1.99 to 15.55)	1.59 (1.12 to 2.25)	1.58 (1.10 to 2.26)	.01
≥3 episodes of hypoglycemia	8/34 (23.5)	8.64 (-6.18 to 23.45)	1.58 (0.82 to 3.06)	1.33 (0.69 to 2.55)	.39

Abbreviations: RD, risk difference; CI, confidence interval; RR, risk ratio.

Excluded 604 children randomized to prophylactic buccal dextrose gel (229 in the hypoglycemia group and 375 in the normoglycemia group).

[a] Generalized linear models adjusted for study site, primary reason for risk of hypoglycemia, socioeconomic decile at birth and multiple births.

[b] Normoglycemia was defined as all blood glucose concentrations ≥47 mg/dL.

[c] An episode of hypoglycemia was defined as ≥1 episode of consecutive blood glucose concentrations of <47 mg/dL.

[d] Mild hypoglycemia was defined as ≥1 episode of ≥36 and <47 mg/dL.

[e] Severe hypoglycemia was defined as ≥1 episode of <36 mg/dL.

© 2022 Edwards T et al. *JAMA Network Open.*
